# Vaccinia virus for lung cancer therapy: preclinical progress and prospects as a systemic immunotherapy platform

**DOI:** 10.3389/fimmu.2026.1797922

**Published:** 2026-05-07

**Authors:** Linkai Li, Zhongyu Chen, Yunrui Yan, Lening Zhang

**Affiliations:** Department of Thoracic Surgery, China-Japan Union Hospital of Jilin University, Changchun, Jilin, China

**Keywords:** immunotherapy, lung cancer, oncolytic virotherapy, preclinical and clinical studies, tumormicroenvironment, vaccinia virus

## Abstract

Lung cancer remains a major therapeutic challenge, particularly when tumors resist targeted therapy or checkpoint blockade. Vaccinia virus (VV), historically used as a vaccine vector, is now being repurposed to selectively kill tumor cells while activating antitumor immunity. Beyond direct oncolysis, VV induces inflammatory cell death that recruits dendritic cells and reshapes tumor–immune interactions—a critical advantage in the immune-suppressed lung tumor microenvironment. This review highlights evidence from lung cancer models, detailing how specific VV strains modulate immunity and under which conditions these effects are therapeutically relevant. We discuss engineering strategies, from cytokine expression to enhanced costimulation, designed to convert “cold” tumors into responsive ones and enable rational combinations with checkpoint inhibitors. Early clinical data in non–small cell lung cancer are promising but also reveal key obstacles: inefficient systemic delivery and rapid immunosuppressive feedback within the tumor. Addressing these challenges will be essential for establishing VV as a robust systemic immunotherapy platform.

## 1 Introduction

Lung cancer remains the world’s deadliest malignancy. In 2022 alone, more than 2.5 million people were diagnosed, with China accounting for over one million deaths—an imbalance that reflects both global prevalence and regional urgency (1–3). Most cases are non-small cell lung cancer (NSCLC), where targeted drugs and immune checkpoint inhibitors have altered clinical practice yet improved survival only modestly; long-term outcomes still hover below 30% (4–8). Small-cell lung cancer (SCLC), despite its initial chemosensitivity, continues to relapse early, and durable systemic options remain scarce (9, 10). Together, these realities underscore an unmet need for treatments that extend beyond transient control.

Immunotherapy has shifted the therapeutic landscape, but its benefits are inconsistent. Checkpoint inhibitors help only a subset of patients, with limited responses and frequent immune escape despite promising biomarkers such as PD-L1 expression and high tumor mutational burden (11–14). As a result, the focus is moving toward strategies that not only kill tumor cells but actively reshape the tumor microenvironment to sustain meaningful, long-term immune control (15–18).

Oncolytic viruses (OVs) have gained prominence for this dual role. By selectively infecting and lysing malignant cells, they trigger immunogenic cell death and convert the tumor into a source of antigens—effectively functioning as an *in situ* vaccine (19, 20). The FDA approval of T-VEC validated this concept clinically and accelerated exploration of new viral platforms (19, 21, 22).

Vaccinia virus (VV) stands out among OVs because of its large engineering capacity, cytoplasmic replication, and long-established safety record (23, 24). Beyond direct tumor lysis, VV possesses the unique ability to modulate the tumor microenvironment, enhance antigen presentation, and amplify systemic immune responses, making it particularly well suited for combination with immune checkpoint blockade and other immunotherapies (25).

Rather than viewing VV solely as a cytolytic oncolytic virus, we propose that it should be conceptualized as a programmable immunotherapy platform. In lung cancer—where immune exclusion, heterogeneous antigen expression, and adaptive resistance limit durable responses—VV-based strategies may exert their greatest therapeutic impact through systemic immune activation and microenvironmental reprogramming rather than direct oncolysis alone. Accordingly, this review is structured around this conceptual framework. We examine preclinical and clinical evidence through the lens of immune mensic modulation, delivery optimization, and rational combination strategies, and discuss how VV may evolve into a modular platform designed to support durable and personalized systemic therapy.

## 2 Mechanistic basis of vaccinia virus–mediated antitumor immunity

### 2.1 Intrinsic biological features of vaccinia virus relevant to antitumor immunity

**Figure 1:**
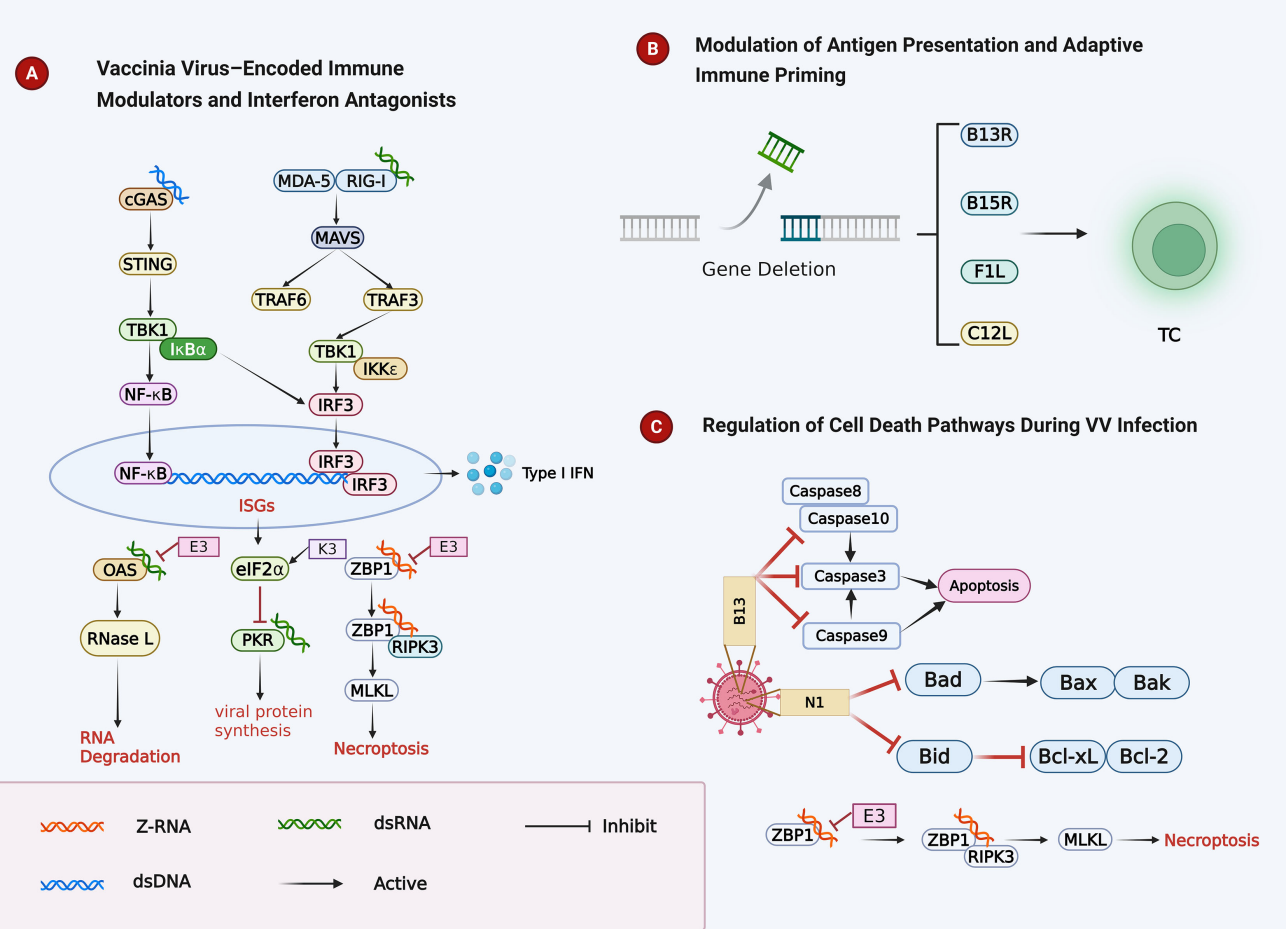
VV, vaccinia virus modulation of innate immunity, antigen presentation, and cell death pathways. **(A)** VV proteins antagonize cytosolic nucleic acid sensing pathways (cGAS–STING and RIG-I/MDA5–MAVS), inhibiting IRF3/NF-κB activation, type I IFN production, and ISG-mediated antiviral responses. **(B)** Deletion of immunomodulatory genes (B13R, B15R, F1L, C12L) enhances T cell activation by improving antigen presentation and immune priming. **(C)** VV inhibits apoptosis and necroptosis through viral factors targeting caspases, Bcl-2 family proteins, and the ZBP1–RIPK3–MLKL pathway.

The intrinsic biological features of vaccinia virus (VV) relevant to antitumor immunity, including cytoplasmic replication, immune evasion, modulation of antigen presentation, and cell death regulation, are summarized in **Figure 1**.

#### 2.1.1 Cytoplasmic replication and innate immune sensing dynamics

Vaccinia virus (VV) is a large double-stranded DNA virus that completes its entire replication cycle in the cytoplasm, encoding its own transcriptional and replication machinery (26, 27). Gene expression proceeds in early, intermediate, and late phases under multistage promoter control, allowing precise temporal regulation of viral protein production (28). Many immune modulators are expressed during the early phase, before genome replication, ensuring rapid establishment of an antiviral-resistant intracellular environment (29).

Cytoplasmic replication inherently generates immunostimulatory nucleic acid intermediates, including dsRNA and Z-RNA, which are sensed by PRRs such as RIG-I/MDA5, cGAS, and ZBP1 (26, 27). Activation of these pathways triggers type I interferon production and inflammatory cell death. Thus, VV replication simultaneously exposes potent PAMPs while deploying early countermeasures to restrain innate sensing.

For oncolytic applications, this replication strategy enables rapid intratumoral amplification coupled with dynamic engagement of innate immune pathways (30) (**Figure 1**).

#### 2.1.2 Vaccinia virus–encoded immune modulators and interferon antagonists

Approximately one-third to one-half of the VV genome is devoted to immune evasion a (26, 29). Central to IFN resistance are E3L and K3L.

E3L encodes a multifunctional protein with a C-terminal dsRNA-binding domain and an N-terminal Zα domain (27). By sequestering dsRNA, E3 blocks PKR activation and OAS/RNase L signaling, thereby preventing translational shutoff and RNA degradation (27, 31). E3 also suppresses RNA polymerase III– and cGAS-dependent IFN-β induction (26). Importantly, its Zα domain binds viral Z-RNA to prevent ZBP1-mediated necroptosis; disruption of this domain results in rapid RIPK3–MLKL activation and marked *in vivo* attenuation (27).

K3L encodes an eIF2α mimic that competitively inhibits PKR, preserving viral protein synthesis under IFN pressure (27).

Extracellular immune evasion further enhances viral persistence. B18R functions as a secreted type I IFN decoy receptor that neutralizes IFN-α/β and limits paracrine antiviral signaling (30). Deletion of B18R enhances tumor selectivity and immunotherapeutic potency.

VV also resists complement-mediated neutralization. The extracellular enveloped virion (EEV) form incorporates host complement regulatory proteins such as CD55, CD46, and CD59, conferring complement resistance (32). Engineering complement control protein expression further enhances systemic stability (32).

Collectively, VV integrates intracellular PAMP masking with extracellular cytokine and complement evasion to balance replication competence and immune visibility (**Figure 1**).

#### 2.1.3 Modulation of antigen presentation and adaptive immune priming

Despite extensive immune evasion, VV infection frequently induces robust T cell responses.

Viral replication culminates in tumor cell lysis and release of tumor antigens and danger signals, facilitating dendritic cell (DC) uptake and cross-presentation to CD8^+^ T cells (30). The nature of cell death critically influences adaptive priming: apoptotic death is relatively immunologically silent, whereas necroptosis promotes coordinated CD4^+^ and CD8^+^ T cell activation through DAMP/PAMP release (29).

Deletion of specific immunomodulatory genes (e.g., B13R, B15R, F1L, C12L) enhances CD8^+^ T cell magnitude and memory formation, demonstrating that viral immune evasion proteins constrain adaptive immunity (26, 29).

The thymidine kinase (TK) locus, a non-essential gene region, provides a stable insertion site for transgenes (33). TK deletion attenuates replication in normal tissues while preserving tumor selectivity, forming the backbone of many oncolytic VV platforms.

Thus, VV can function as an *in situ* vaccine platform, linking direct oncolysis to adaptive immune priming (**Figure 1**).

#### 2.1.4 Regulation of cell death pathways during VV infection

Regulation of host cell death is central to VV pathobiology and therapeutic immunogenicity.

VV encodes multiple anti-apoptotic proteins expressed early during infection (29). B13 (SPI-2) is a potent broad-spectrum caspase inhibitor targeting caspase-1, -8, -9, and -10; F1L and N1 adopt Bcl-2–like folds and inhibit mitochondrial apoptosis (34, 35). These proteins delay intrinsic and extrinsic apoptosis to allow completion of viral replication.

When caspase activity is suppressed, necroptosis may emerge as a backup antiviral response. VV-induced programmed necrosis involves RIPK1/RIPK3 complex formation, MLKL activation, HMGB1 release, ATP depletion, and metabolic collapse (36). Importantly, necroptosis induction can occur early and independently of TNF-α signaling (36).

E3-mediated binding of Z-RNA prevents ZBP1-driven necroptosis; mutation of the Zα domain triggers rapid inflammatory cell death and reduces viral fitness (27).

Overall, VV manipulates apoptosis and necroptosis in a temporally coordinated manner: early suppression preserves replication, whereas eventual inflammatory death enhances antigen release and immune activation (**Figure 1**).

### 2.2 Tumor-directed effects of vaccinia virus

**Figure 2:**
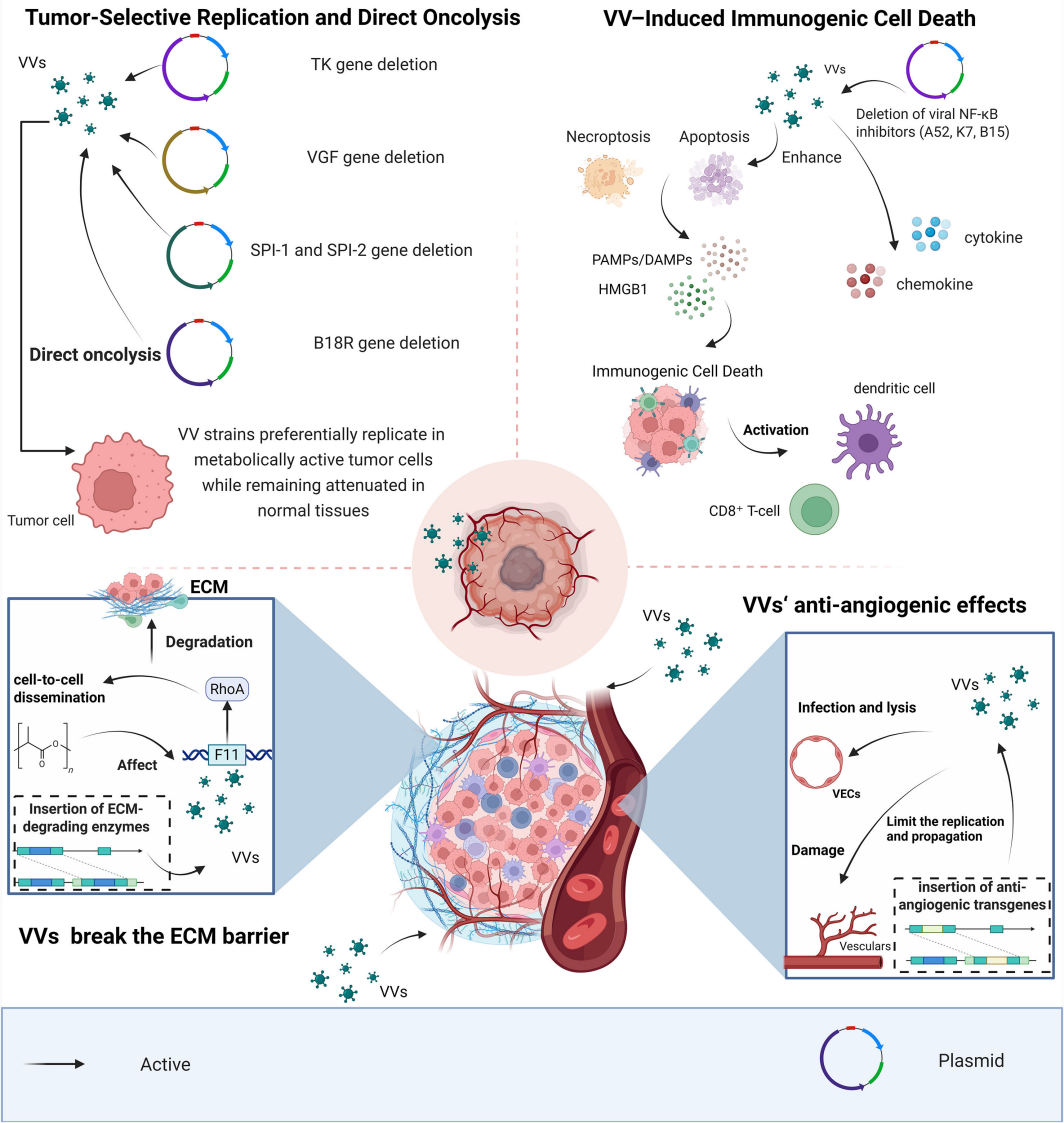
Antitumor mechanisms of oncolytic VVs, vaccinia viruses. VVs selectively replicate in metabolically active tumor cells through deletion of genes such as TK, VGF, SPI-1/SPI-2, and B18R, resulting in direct oncolysis. VV-induced apoptosis and necroptosis promote immunogenic cell death, leading to release of PAMPs/DAMPs, cytokines, and chemokines that activate dendritic cells and CD8^+^ T cells. VVs also enhance intratumoral spread by degrading the ECM, extracellular matrix and can inhibit tumor angiogenesis through infection of vascular endothelial cells or insertion of anti-angiogenic transgenes.

The antitumor mechanisms of oncolytic vaccinia viruses (VVs), including tumor-selective replication, immunogenic cell death, stromal remodeling, and vascular modulation, are summarized in **Figure 2**.

#### 2.2.1 Tumor-selective replication and direct oncolysis

Tumor-selective replication constitutes the primary mechanism underlying direct oncolysis by vaccinia virus (VV). Engineered VV strains achieve conditional replication by coupling viral amplification to tumor-associated metabolic activity, oncogenic signaling, and defects in antiviral responses.

Deletion of the viral thymidine kinase (TK) gene is the most widely used strategy to confer tumor selectivity (33, 37, 38). While viral TK supports nucleotide synthesis in quiescent cells, proliferating tumor cells typically exhibit elevated endogenous TK activity and upregulated nucleotide salvage pathways, thereby compensating for viral TK loss. Consequently, TK-deficient VV strains preferentially replicate in metabolically active tumor cells while remaining attenuated in normal tissues. Clinically advanced constructs such as JX-594 (Pexa-Vec) are based on this principle (39).

Tumor selectivity can be further reinforced by deleting vaccinia growth factor (VGF), which limits replication in cells lacking constitutively activated EGFR–Ras signaling pathways—frequently dysregulated in epithelial malignancies (39). Double-deleted strains (e.g., TK^-^/VGF^-^ vvDD) thus rely more strictly on tumor-intrinsic proliferative signaling for efficient replication.

Additional selectivity layers derive from modulation of host cell death and interferon pathways. Vaccinia encodes anti-apoptotic serpins such as SPI-1 and SPI-2; their deletion renders the virus more susceptible to apoptosis-mediated restriction in normal cells, while replication may remain permissive in tumors with dysregulated cell death control (40). Similarly, deletion of B18R, a viral type I interferon–binding protein, can enhance attenuation in interferon-competent normal tissues, although this effect is context-dependent and varies with tumor antiviral competence (41).

Collectively, these strategies do not confer absolute specificity but generate a probabilistic replication bias toward malignant tissue. By preferentially replicating in cells exhibiting hallmark features of cancer—including hyperproliferation, oncogenic signaling autonomy, apoptosis dysregulation, and impaired antiviral responses—engineered VV platforms establish a conditional replication phenotype that culminates in direct cytolysis within the tumor microenvironment (**Figure 2**).

#### 2.2.2 Vaccinia virus–induced immunogenic cell death

In addition to direct cytolysis, vaccinia virus (VV) infection can induce tumor cell death with immunogenic features. Certain VV strains trigger programmed necrosis or mixed apoptotic–necrotic phenotypes accompanied by release of danger-associated molecular patterns (DAMPs). In ovarian cancer models, Lister strain VV induced programmed necrosis with extracellular HMGB1 release (36). TK-deficient and vvDD variants have also been reported to promote ATP and HMGB1 release and calreticulin exposure—molecular hallmarks commonly associated with immunogenic cell death (ICD) (40). However, fulfillment of functional ICD criteria, including efficient dendritic cell cross-priming, appears model- and strain-dependent.

VV-induced cell death is coupled to inflammatory amplification. Viral sensing activates NF-κB–dependent cytokine and chemokine production, promoting immune cell recruitment (42). Deletion of viral NF-κB inhibitors (A52, K7, B15) in the NYVAC backbone enhances this inflammatory program, underscoring the importance of viral immunomodulatory gene composition in shaping tumor-associated inflammation (42).

Functionally, VV-mediated lysis enables concurrent release of tumor antigens and inflammatory signals, supporting dendritic cell activation and CD8^+^ T-cell priming (43, 44). In preclinical models, tumor eradication is frequently associated with resistance to rechallenge, consistent with adaptive immune memory formation (41). Overall, VV-induced immunogenic cell death may represent a context-dependent bridge between local oncolysis and systemic antitumor immunity (**Figure 2**).

#### 2.2.3 Impact of vaccinia virus on tumor stroma and extracellular matrix: evidence and knowledge gaps

Direct VV-specific evidence demonstrating active remodeling of tumor stroma or enzymatic degradation of extracellular matrix (ECM) components remains limited. Although vaccinia virus (VV) exhibits efficient intratumoral spread, available data suggest that this property primarily reflects robust cytoplasmic replication and modulation of cytoskeletal dynamics rather than direct matrix degradation. For example, the viral F11 protein enhances cell-to-cell dissemination through regulation of RhoA signaling; however, this mechanism influences intracellular trafficking and junctional architecture, not stromal ECM breakdown (39, 45, 46). Systematic investigations of VV effects on collagen deposition, cancer-associated fibroblasts, or desmoplastic barriers are currently sparse.

Proposed strategies to enhance stromal penetration—such as insertion of ECM-degrading enzymes—are largely extrapolated from broader oncolytic virus engineering concepts (37). While biologically plausible, published data demonstrating functional ECM-degrading transgenes in vaccinia backbones remain limited. Thus, stromal remodeling should presently be regarded as a potential optimization strategy rather than an established VV-intrinsic mechanism.

Comparative evidence from non-VV platforms provides important context. Adenoviral vectors expressing hyaluronidase have shown improved dispersion in matrix-dense tumors, highlighting the feasibility of enzymatic ECM targeting in other systems. In contrast, VV appears to retain replication competence under hypoxic conditions where adenoviral protein synthesis is impaired (47), suggesting that its relative advantage may derive from replication robustness within hostile microenvironments rather than active stromal degradation (**Figure 2**).

Overall, the impact of VV on tumor stroma remains incompletely characterized, and mechanistic delineation of stromal interactions represents a key area for future investigation.

#### 2.2.4 Vascular and angiogenic modulation by vaccinia virus

Clinical and preclinical observations indicate that vaccinia virus (VV) infection can be associated with rapid disruption of tumor perfusion, contributing to extensive tumor necrosis beyond direct tumor cell lysis (48). Studies involving TK-deleted constructs such as JX-594 (Pexa-Vec) report early reductions in tumor blood flow following intratumoral administration (49). Importantly, these findings primarily reflect replication-associated vascular collapse rather than a defined, VV-encoded anti-angiogenic gene program. The underlying mechanisms likely involve a combination of intratumoral viral amplification, inflammatory cytokine release, endothelial stress, and secondary thrombosis. Direct evidence for consistent infection and lysis of tumor-associated endothelial cells, however, remains limited.

Engineering approaches have explored insertion of anti-angiogenic transgenes into VV backbones (39–41). Nevertheless, compared with replication-driven vascular disruption, transgene-mediated angiogenic inhibition in vaccinia platforms remains less extensively validated at the clinical level. Combination strategies, such as Pexa-Vec with VEGF pathway inhibitors (e.g., sorafenib), further underscore that pharmacologic angiogenesis blockade often complements rather than derives intrinsically from VV biology (39).

By comparison, other oncolytic platforms—including HSV-based vectors such as T-VEC—are primarily engineered to enhance immunostimulatory transgene expression rather than to induce vascular collapse (50, 51). Thus, while VV may exert context-dependent vascular-disruptive effects, these appear largely secondary to replication dynamics and tumor inflammation rather than representing a broadly defined anti-angiogenic mechanism (52) (**Figure 2**).

Taken together, VV-associated vascular modulation should be interpreted as a replication-coupled phenomenon with variable clinical expression, and its precise cellular basis warrants further mechanistic clarification.

### 2.3 Mechanistic limitations and context dependency

The antitumor efficacy of vaccinia virus (VV) is highly context-dependent, influenced by viral, host, and tumor-specific factors.

#### 2.3.1 Mechanistic model dependence

VV efficacy reflects a balance between direct oncolysis and immune-mediated responses. Premature immune activation can clear the virus too early, reducing oncolysis, while reliance solely on oncolysis may be insufficient. Temporal control of transgene expression allows switching from oncolytic to immunostimulatory modes (53). Moreover, the type of immunogenic cell death (apoptosis, necroptosis, pyroptosis) dictates the magnitude of immune activation (54).

#### 2.3.2 Immune-competent versus immune-deficient models

Therapeutic outcomes are strongly dependent on host immunity. In immune-deficient mice, VV plus immune effectors fail to clear tumors, whereas in immune-competent hosts, they induce durable antitumor immunity (30). Similarly, combination therapies, including immune checkpoint blockade, require intact adaptive immunity (35).

#### 2.3.3 Replication-competent VV versus non-replicating MVA

Mechanisms differ fundamentally between replication-competent VV and highly attenuated, non-replicating vectors such as MVA or NYVAC. Replicating VV mediates oncolysis and functions as an *in situ* vaccine, whereas non-replicating vectors primarily deliver antigens. Importantly, immune activation can occur independently of viral replication, for example via NF-κB activation following targeted viral gene deletions (39, 42, 55).

#### 2.3.4 Tumor-type specific variability

VV cytotoxicity and immune stimulation vary by tumor type and microenvironment. Lister strain VV predominantly induces programmed necrosis in ovarian cancer cells, with cell line–dependent and hypoxia-modulated effects (36, 47). Tumor immunogenicity further modulates outcomes, as highly immunogenic cancers respond more robustly to VV-mediated therapies, whereas immunosuppressive microenvironments may favor viral replication but limit immune activation (56).

#### 2.3.5 Transgene versus intrinsic viral effects

Separating intrinsic viral effects from those conferred by engineered transgenes is critical. Controlled transgene expression and gene deletion/complementation studies demonstrate that intrinsic viral oncolysis and immune activation can be distinguished from the additional effects provided by transgenes such as GM-CSF or IL-2 (39, 57, 58).

VV efficacy is shaped by viral strain, replication competency, host immunity, tumor type, and transgene contribution. These mechanistic considerations highlight the importance of interpreting VV-induced immune effects within specific tumor contexts, which will be further explored in Chapter 6.

## 3 Platform-level advantages of vaccinia-derived platforms for lung cancer translation

Vaccinia-derived vectors used in cancer therapy comprise two biologically distinct categories: (1) replication-competent vaccinia virus (VV) constructs designed for oncolytic activity, and (2) highly attenuated, non-replicating derivatives such as Modified Vaccinia Ankara (MVA) and NYVAC.

Given their divergent replication capacity and translational roles, these platforms are considered separately.

VV Construct Nomenclature and Notation Framework.

In this review, we preserve the original nomenclature of all vaccinia virus (VV) constructs as reported in the cited studies. To facilitate comparison and understanding, we introduce a standardized notation framework describing the construct backbone, gene deletions, payloads, and delivery routes. For example:

- Backbone indicates the parental VV strain (e.g., WR, Copenhagen, Lister).
- Deletions are indicated with Δ followed by gene symbols (e.g., ΔTK).
- Payloads represent transgenes or expressed proteins (e.g., IL-2, GFP).
- Delivery route is provided in parentheses (e.g., IV, IT, pleural).

Using this framework, readers can readily interpret each construct while retaining the original nomenclature from the literature.

### 3.1 Genome architecture and design flexibility

#### 3.1.1 Large genome and multi-transgene capacity

Vaccinia virus possesses a ~190 kb double-stranded DNA genome capable of accommodating at least 25 kb—and in some reports up to ~40 kb—of foreign DNA (43, 59, 60).This large genomic capacity enables simultaneous insertion of multiple therapeutic elements within a single vector, including cytokines, tumor antigens, costimulatory molecules, or reporter genes (61, 62).

Importantly, this multi-transgene permissiveness is an intrinsic structural property of VV and does not depend on tumor type, making it broadly applicable to lung cancer engineering strategies.

#### 3.1.2 Defined insertion loci and modular genome editing

The thymidine kinase (TK) locus, molecularly mapped by Hruby and Ball (1982), represents a canonical non-essential insertion site. Additional loci such as hemagglutinin (HA) have also been utilized (42).More than 20 immunomodulatory genes have been identified as genetically modifiable (63), providing multiple entry points for rational genome editing. Rather than reflecting intrinsic tumor biology (discussed in Chapter 2), these loci function as modular engineering sites, allowing customizable vector construction.

#### 3.1.3 Conditional replication as a design strategy

Deletion of genes such as TK or VGF has been widely employed to create tumor-biased replication platforms (64, 65).At the platform level, such deletions should be viewed as conditional design modules that can be combined with transgene insertion, immune modulation, or safety attenuation strategies. The emphasis here is not the biological mechanism of selectivity (addressed in Chapter 2), but the modular nature of VV genome editing that enables layered engineering logic.

### 3.2 Translational advantages as a therapeutic platform

#### 3.2.1 Genetic engineering flexibility

A substantial proportion of VV genes are non-essential and many encode immunomodulatory proteins (66, 67).Their targeted deletion or modification permits tuning of viral immunogenicity and inflammatory output. For example, simultaneous deletion of NF-κB inhibitory genes (A52R, K7R, B15R) in NYVAC demonstrated feasibility of multi-gene reprogramming (42).This combinatorial manipulability distinguishes VV as a programmable vector rather than a fixed biological entity.

#### 3.2.2 Virion diversity and dissemination properties

VV naturally produces intracellular mature virus (IMV) and extracellular enveloped virus (EEV), the latter facilitating systemic dissemination and partial complement resistance (43). At the translational level, this dual-particle biology provides a structural basis for exploring both intratumoral and systemic administration strategies, particularly relevant to metastatic lung cancer.

#### 3.2.3 Clinical safety legacy

Vaccinia virus has an unparalleled history of human use in smallpox eradication (68).This extensive clinical familiarity, together with established antiviral countermeasures, contributes to regulatory confidence and facilitates translational development (37, 69).This historical safety framework represents a platform-level advantage distinct from mechanistic tumor interactions.

#### 3.2.4 Adaptability to hypoxic microenvironments

Under hypoxic conditions (1% O_2_), Lister strain VV maintains replication competence and cytotoxicity (47).Although these data are not lung-exclusive, they suggest biological robustness in oxygen-variable environments. At the platform level, this property supports consideration of VV in structurally heterogeneous lung tumors, pending tumor-specific validation.

In brief, vaccinia virus combines a large, flexible genome, modular engineering sites, virion diversity, and established clinical safety, making it a robust and adaptable platform for lung cancer therapeutics.

## 4 Preclinical advances of vaccinia virus in lung cancer therapy

Oncolytic virotherapy has grown into a notable branch of cancer immunotherapy, and vaccinia virus (VV) is frequently regarded as one of the more practical candidates to push toward clinical translation. Interest in VV is not accidental: decades of documented human use provide reassurance on safety, and recent investigations have started to reveal meaningful antitumor performance in lung cancer settings, especially in non-small cell lung cancer (NSCLC) (19).

Given the biological and functional differences between replication-competent vaccinia constructs and non-replicating modified vaccinia Ankara (MVA) platforms, this chapter distinguishes these strategies in separate subsections to clarify their respective mechanisms and therapeutic implications. Where applicable, findings derived specifically from lung cancer models are explicitly indicated, whereas data informed by studies in other solid tumors are identified as such to avoid overgeneralization.

Current research efforts tend to cluster around two directions. One involves designing more refined viral backbones— most commonly replication-competent VV constructs-typically by introducing therapeutic genes that heighten tumor selectivity or strengthen immune activation. The other focuses on integrating VV or MVA-based platforms with existing cancer therapies, such as checkpoint blockade or standard chemotherapeutics, to harness potential synergistic effects (70).

Meanwhile, researchers are working on delivery strategies tailored to the complexity of lung cancer biology. These approaches aim to overcome challenges including the limited viral penetration into metastatic niches and the immunological inertia of so-called “cold” tumors (71). Together, they illustrate that progress is not solely about making “better viruses,” but also about enabling those viruses to reach and sustain activity within difficult tumor ecosystems.

The remainder of this chapter will highlight representative preclinical advances in VV-based lung cancer therapy, drawing primarily from lung cancer–specific *in vitro* and *in vivo* models while clearly indicating instances where mechanistic insights are extrapolated from other solid tumors.

### 4.1 Antitumor characteristics of replication-competent vaccinia virus *in vitro* lung cancer models” for grammatical consistency

The *in vitro* assays summarized below primarily evaluate replication-dependent cytolytic activity and therefore predominantly involve replication-competent oncolytic VV constructs. Replication-deficient platforms such as Modified Vaccinia Ankara are not optimally assessed using conventional monolayer cytotoxicity models, as their therapeutic function relies more heavily on immune priming mechanisms that are better captured in *in vivo* systems. The *in vitro* studies summarized below primarily involve replication-competent oncolytic VV constructs.

#### 4.1.1 *In vitro* evaluation of viral infectivity and oncolysis

The constructs discussed in this subsection are restricted to those evaluated in lung cancer–specific *in vitro* models unless otherwise specified.

*In vitro* experiments remain a cornerstone for assessing the antitumor potential of vaccinia virus (VV). A typical strategy involves infecting widely used NSCLC cell lines—such as A549, H1299, H1650, and H460—to evaluate both viral entry efficiency and subsequent cytolytic activity. Across multiple studies, VV consistently demonstrates potent oncolytic effects in this diverse set of lung cancer models (43). Of particular interest are engineered VV strains harboring deletions in the thymidine kinase (TK) gene, including GLV-1h68 and GLV-1h151, which achieve high infection rates in A549 cells and induce marked morphological changes along with significant reductions in cell viability (64).

To monitor viral behavior more precisely, reporter genes like GFP or RUC-GFP are often incorporated into the viral genome. These modifications enable real-time visualization of viral replication and the spread of infection from cell to cell within cultured monolayers (72).

Collectively, these *in vitro* approaches provide a robust, reproducible platform for quantifying the lytic capacity of different VV constructs. Importantly, the insights gained from such studies are critical for guiding the design of next-generation VV variants with improved oncolytic performance.

#### 4.1.2 Viral engineering strategies for functional enhancement

To extend the intrinsic therapeutic potential of vaccinia virus, researchers have increasingly relied on targeted genetic engineering. One common approach involves introducing genes that encode proteins capable of boosting viral replication and tumor penetration. For instance, the engineered WCL virus incorporates a gene for white-spotted chondroitin sulfate lectin, a modification that markedly accelerates replication and allows the virus to infiltrate more deeply into three-dimensional NSCLC cell cultures (73). In a similar vein, the VV-CLEC2A construct expresses a C-type lectin protein, which has been shown to enhance both direct tumor cell killing and cell-to-cell viral dissemination (74).

Genetic modifications can also intensify the cytolytic impact on infected cells. Strains such as GLV-1h376 trigger robust oncolysis in lung cancer cells, manifesting as membrane disruption, nuclear condensation, and accumulation of apoptotic debris (75). These results collectively underscore that well-planned genetic interventions can substantially elevate the virus’s native tumor-killing capacity, offering a rational framework for designing next-generation oncolytic VV platforms.

#### 4.1.3 Cell death pathways triggered by oncolytic vaccinia virus

The tumor-killing effects of vaccinia virus (VV) extend well beyond simple membrane disruption, engaging specific programmed cell death mechanisms. In A549 lung cancer cells, studies reveal that infection does not trigger the activation of classical apoptotic markers such as caspase-3, -7, or -8, nor does it induce assembly of the ASC inflammasome. Instead, VV strongly stimulates the RIPK1/RIPK3 signaling pathway, a key mediator of necroptosis, which is a regulated, caspase-independent form of cell death (40). This capacity to induce necroptosis suggests that VV can exploit an alternative lethal pathway, potentially overcoming apoptotic resistance that is frequently observed in poorly immunogenic tumor microenvironments.

These findings suggest that VV can bypass apoptosis resistance in NSCLC cells by inducing alternative death pathways, offering a potential therapeutic advantage particularly in immune-cold tumors.

#### 4.1.4 Immune activation pathways triggered by vaccinia virus

In parallel, significant efforts in viral engineering aim to enhance VV’s immunostimulatory properties. Recombinant constructs are designed to activate immune pathways both directly and indirectly. For example, rVV40L expresses CD40 ligand (CD40L), which not only triggers apoptosis in certain cancer cells but also robustly activates macrophages, improving their antigen-presentation and cytotoxic functions (44).

To further direct adaptive immune responses, VV vectors have been armed with tumor-associated antigens such as MAGE-A4, MAGE-A10, and NY-ESO-1. When combined with the co-stimulatory molecule CD80, these constructs markedly enhance the activation of tumor-specific cytotoxic T lymphocytes (CTLs) (76).

Other sophisticated strategies include encoding bispecific antibodies; EphA2-TEA-VV, for instance, expresses a bispecific EphA2×CD3 antibody that physically links T cells to tumor cells, thereby improving recognition and killing efficiency (38). The multifaceted design of TG6050 exemplifies this approach further, as it co-expresses interleukin-12 (IL-12) and a full-length anti-CTLA-4 antibody, leading to potent CD8^+^ T cell activation *in vitro (*77).

Collectively, these findings illustrate that through targeted genetic modifications, VV has evolved from a conventional oncolytic virus into a versatile immunotherapeutic platform. It can directly eliminate tumor cells via non-apoptotic pathways while simultaneously orchestrating host immune responses, establishing a compelling rationale for its inclusion in combination immunotherapy regimens against lung cancer.

#### 4.1.5 Exploration of mechanistic regulatory pathways

The interaction between vaccinia virus (VV) and lung cancer cells is influenced not only by engineered viral traits but also by the intrinsic signaling networks within the tumor. Recent mechanistic studies have highlighted the c-Jun N-terminal kinase (JNK) pathway as a critical determinant of cellular susceptibility to VV. Specifically, lung cancer cells with low basal JNK activity were observed to support higher levels of viral replication and subsequent cytolysis, whereas activation of the JNK pathway appeared to restrict viral propagation. Mechanistic analysis indicates that this suppression involves upregulation of the protein kinase R (PKR) cascade, a central mediator of the host antiviral response (78).

These findings carry direct implications for therapy. *In vitro* experiments show that pharmacological inhibition of JNK significantly enhances the oncolytic efficacy of VV, suggesting that the pathway could serve as a host-directed target in combination strategies. Such insights provide a compelling rationale for pairing VV-based therapies with small-molecule inhibitors aimed at mitigating tumor-intrinsic antiviral defenses, thereby potentially amplifying therapeutic outcomes.

Overall, these studies collectively shed light on how VV engages lung cancer cells at the molecular level. To provide a structured overview, **Table 1** compiles data on the various VV constructs tested, detailing their genetic modifications alongside the observed cytolytic and immunomodulatory effects in different lung cancer cell lines. This comparative presentation not only facilitates evaluation of distinct engineered variants but also underscores the diverse cellular mechanisms by which VV exerts oncolytic activity. Taken together, the insights gained from these *in vitro* studies establish a strong foundation for the rational design of next-generation VV-based therapeutics.

**Table 1:** Summary of genetically modified vaccinia virus strains evaluated in lung cancer *in vitro* models.

Virus name	Genetic modifications	Model	Antitumor efficacy	PMID
GLV-1h151	LIVP.F14.5L^-^::RUC-GFP/J2R^-^::lacZ/A56R^-^::hNIS^a^	H1650	~50% cell death by day 9 at low MOIs (0.01, 0.1); <20% viability at MOI 1.0	22258815
Western Reserve strain	None	A549	Necroptosis induction;↑LC3-II/LC3-I ratio;No caspase-3/-7/-8 activation;Absence of ASC inflammasome formation;Non-apoptotic/pyroptotic cell death	31969562
VV-WCL	WR.TK^-^::WCL^b^	H460	↑Cytotoxicity & replication in H460;activates caspase-3/9;induces apoptosis	34064193
GLV-1h68	LIVP,ΔF14.5L/J2R/A56R,+RUC-G FP,+LacZ,+GusA^c^	H727, UMC-11	60% cell clearance at an MOI of 0.1;↑virus titer >10^7^ PFU/ml; no antagonism with everolimus	32631270
VV-CLEC2A	WR.TK^-^::CLEC2A^d^	H460	↑Cytotoxicity, pro-apoptotic activity, and viral replication; CLEC2A as immunomodulatory factor^h^	39006946
EphA2-TEA-VV	WR.VSC20(ΔTK,ΔVGF),+EphA2-T EA(scFv×2)^e^	A549, H1299	Encodes EphA2-CD3 bispecific engager; targets tumor and T cells; replication similar to wild-type VV; enables local antibody secretion	24135899
GLV-1h210	GLV-1h68-derived,TK^-^::hEPO(under P7.5E promoter)^f^	A549	Efficient replication, Enhanced cytolysis, hEPO/hEPO(R103A) driven by P7.5E, Time-dependent hEPO accumulation	23765443
vvDD	WR.ΔTK,ΔVGF^g^	MAD109, A549	selective oncolysis, antigen-specific cytotoxicity *in vitro*, enhanced CD4^+^/CD8^+^ T cell activation	32461344

^a^LIVP.F14.5L^-^::RUC-GFP/J2R^-^::lacZ/A56R^-^::hNIS: multi-gene deletion and reporter insertion strain.

^b^WR.TK^-^::WCL: thymidine kinase deletion, WCL insertion.

^c^GLV-1h68: F14.5L/J2R/A56R deletions, RUC-GFP, LacZ, GusA reporters.

^d^WR.TK^-^::CLEC2A: TK deletion, CLEC2A immunomodulatory insertion.

^e^EphA2-TEA-VV: ΔTK, ΔVGF deletions, EphA2-CD3 bispecific engager.

^f^GLV-1h210: derived from GLV-1h68, TK deletion, hEPO under P7.5E promoter.

^g^vvDD: WR TK, VGF deletions.

^h^↑, increase or enhancement; ↓, decrease or reduction.

TK, thymidine kinase; GFP, green fluorescent protein; LacZ, β-galactosidase; hNIS, human sodium iodide symporter; RUC, Renilla luciferase; GusA, β-glucuronidase; hEPO, human erythropoietin; P7.5E, P7.5 early promoter; MOI, multiplicity of infection; LC3-II, lipidated form of microtubule-associated protein 1A/1B-light chain 3; CLEC2A, C-type lectin domain family 2 member A; CD4^+^/CD8^+^ T cells, CD4-positive and CD8-positive T lymphocytes; ASC, apoptosis-associated speck-like protein containing a CARD; LC3-I, non-lipidated form of microtubule-associated protein 1A/1B-light chain 3; A549, H1650, H727, UMC-11, H460, MAD109, respective lung cancer cell lines.

### 4.2 VV-based therapeutic strategies in lung cancer animal models: from oncolytic effects to immune modulation

*In vivo* investigations using lung cancer models have provided strong evidence of the dual oncolytic and immunostimulatory potential of replication-competent oncolytic vaccinia virus (VV) constructs. In contrast to non-replicating modified vaccinia Ankara (MVA)–based vaccine platforms, which primarily function through antigen delivery and immune priming (discussed in Section 5), the VV systems summarized here retain the capacity for tumor-selective replication and intratumoral amplification. This replicative competence enables coordinated direct cytolysis and secondary immune activation within the tumor microenvironment.

Across orthotopic, metastatic, and pleural dissemination models, replication-competent VV constructs consistently demonstrate multifaceted antitumor activity. Reported outcomes include direct tumor cell lysis, suppression of metastatic progression, and modulation of the immunosuppressive tumor microenvironment (TME). Importantly, these effects are context-dependent and often arise from the interplay between viral replication dynamics and host immune engagement rather than from viral cytotoxicity alone.

The following subsections therefore focus specifically on replication-competent VV backbones and their engineered derivatives evaluated in animal models. Emphasis is placed on how rational genetic modifications—ranging from single cytokine arming to multi-gene immunomodulatory designs—reshape the balance between viral propagation, safety, and systemic antitumor immunity. Collectively, these studies illustrate the translational potential of oncolytic VV platforms while also highlighting the biological variables that may influence their *in vivo* performance. Unless otherwise specified, the data summarized below are derived from lung cancer–specific murine models.

#### 4.2.1 Foundational modification: immune-stimulatory effects of ΔTKΔN1L-backbone viruses

The double-deletion mutant VVΔTKΔN1L, which lacks both thymidine kinase (TK) and the immune regulator N1L, has emerged as a well-characterized foundational platform for oncolytic therapy. In Lewis lung carcinoma (LLC) models, this virus exhibited a favorable safety profile. Although its effects on primary tumor growth were modest, treatment markedly decreased the development of pulmonary metastases and improved overall survival. Mechanistically, these systemic benefits were associated with robust activation of natural killer (NK) cells and pronounced upregulation of pro-inflammatory cytokines, including IL-1α, IL-1β, G-CSF, and CXCL1 (64). These findings highlight the backbone virus’s intrinsic potential to target micro metastatic disease and underscore the importance of baseline immunostimulation as a foundation for further therapeutic enhancement.

#### 4.2.2 Functional enhancement: IL-12 arming to boost immunostimulatory capacity

Building on these foundational effects, the VVΔTKΔN1L platform has been further engineered to express interleukin-12 (IL-12), generating VVΔTKΔN1L-IL12. In both LLC and CMT64 lung cancer models, this IL-12-armed virus not only inhibited primary tumor growth but also promoted extensive remodeling of the tumor microenvironment (TME). Observed changes included increased infiltration of cytotoxic CD8^+^ T cells, M1-polarized macrophages, and dendritic cells, alongside a reduction in regulatory T cell populations and suppression of angiogenesis, as indicated by decreased CD31^+^ vessel density. Collectively, these effects contributed to the conversion of immunologically “cold” tumors into “hot” ones (64).

Further refinements, exemplified by the VVL-m12 construct employing a synthetic promoter to control IL-12 expression, enhanced these immunostimulatory effects. Beyond TME remodeling, VVL-m12 treatment facilitated the generation of central memory T (Tcm) cells, suggesting the establishment of a more durable, systemic antitumor immune response capable of long-term surveillance (25). These observations underscore the substantial therapeutic advantage of cytokine arming in oncolytic VV strategies.

#### 4.2.3 Harnessing multi-gene vaccinia viruses to rewire the tumor microenvironment

Moving beyond single-gene modifications, recent efforts have focused on engineering oncolytic VV constructs capable of delivering multiple immunomodulatory factors simultaneously, with the goal of achieving synergistic reprogramming of the tumor microenvironment (TME). One notable example is TG6050, a dual-armed virus designed to co-express interleukin-12 (IL-12) and a fully humanized anti-CTLA-4 monoclonal antibody. In Lewis lung carcinoma (LLC) tumor-bearing mice, TG6050 outperformed single-function vectors, enhancing intratumoral CD8^+^ T cell responses, shifting macrophages toward a pro-inflammatory M1 phenotype, reducing regulatory T cell (Treg) infiltration, and markedly extending overall survival (77).

Similarly, the vB7/β/IL-12 construct combines the T cell costimulatory ligand B7–1 with IL-12. In models of established lung metastases, this multi-factor strategy achieved striking efficacy, suppressing more than 95% of metastatic lesions. These results highlight the potent synergy that arises from simultaneously providing both a critical costimulatory signal and a strong pro-inflammatory stimulus (79).

The principle is further exemplified by VVL-GL21, which co-expresses granulocyte-macrophage colony-stimulating factor (GM-CSF) and IL-21. In CMT64 lung cancer models, this combination induced comprehensive remodeling of the TME, promoting robust infiltration and activation of CD8^+^ T cells and dendritic cells while fostering durable, long-term antitumor immune memory (80).

Collectively, these multi-factor constructs illustrate the therapeutic potential of coordinated gene arming, offering a rational strategy for converting immunologically “cold” tumors into highly immunogenic, treatment-responsive environments.

#### 4.2.4 Xenograft models: dissecting VV mechanisms under varied immune contexts

The antitumor activity of vaccinia virus (VV) depends strongly on the host’s immune context. In immunodeficient mice bearing TC-1 tumors, VV.mIFNβ acted mainly through direct oncolysis, supported by robust viral replication and local interferon-beta (IFNβ) production. By contrast, in immune-competent LKRM2 tumors, efficacy relied primarily on adaptive immunity, with CD8^+^ T cells controlling tumor growth despite limited viral spread (41).

Engineered strategies can further shape the immune environment. VV.CXCL11 recruits antigen-specific T cells into tumors, enhancing infiltration (37).

EphA2-TEA-VV uses a bispecific antibody to bridge T cells and tumor cells, improving local killing and triggering systemic antitumor responses, reducing lung metastases (38, 81).

These findings illustrate that VV can switch between direct cytotoxicity and immune-mediated mechanisms depending on the tumor milieu, highlighting its versatility for rational viral engineering.

#### 4.2.5 Model refinement: efficacy in complex pathological scenarios

Vaccinia virus (VV) therapies have demonstrated potential in advanced, clinically relevant models. In a pleural dissemination model, intrapleural delivery of VV-IL-2 inhibited tumor spread and malignant effusion formation, accompanied by increased TCR clonality and CD8^+^ T cell infiltration, suggesting translational potential for managing pleural metastases (82).

Systemic immune activation from local treatment was observed with vvDD-mIL2 in bilateral LLC tumors, where administration at one site triggered immune responses at distant, untreated tumors (83). Similarly, WR.TK-HPGD^+^ reduced immunosuppressive myeloid-derived suppressor cells (MDSCs) in the tumor microenvironment, enhancing CD8^+^ T and NK cell activity alongside elevated inflammatory cytokines (84).

Targeted immune modulation is further exemplified by vvDD-IL-9, which boosted a specific CCR6^+^ CD8^+^ T cell subset to better suppress metastasis, and vvDD-IL-2-RG, which maintained strong antitumor efficacy while reducing IL-2–associated systemic toxicity (85, 86). Collectively, these studies highlight the versatility of engineered VV in addressing complex tumor pathologies through context-dependent mechanisms.

#### 4.2.6 Exploring the performance and design of vaccinia virus therapies in lung cancer

In preclinical studies of lung cancer, two engineered vaccinia virus (VV) platforms have emerged as representative systems: the GLV-1h68 series and the vvDD vectors. Each platform reflects a distinct design philosophy and offers unique therapeutic strengths.

The GLV-1h68 platform, derived from the LIVP strain, is notable for its capacity to accommodate multiple functional modifications. In PC14PE6-RFP xenograft models, GLV-1h68 induced upregulation of inflammatory mediators such as MCP-1/5 and TNF-α, and its antitumor effects were further enhanced by cyclophosphamide, which improved viral dissemination and tumor control (87). Successive derivatives expanded these capabilities. GLV-1h108, armed with the anti-angiogenic gene *glaf-1*, targeted tumor vasculature effectively (88). GLV-1h151 incorporated the RUC-GFP reporter and human sodium iodide symporter (hNIS), enabling real-time optical and radionuclide imaging of viral distribution while maintaining therapeutic activity (89). GLV-1h210, engineered to express human erythropoietin (hEPO), not only suppressed tumor growth in A549 xenografts but also alleviated cancer-related anemia and improved viral spread through enhanced vascular permeability (90). The progressive engineering of GLV-derived vectors is summarized in **Supplementary Figure S1**.

The vvDD platform, in contrast, emphasizes tumor-selective replication and safety. Its design involves double deletions of the thymidine kinase (TK) and vaccinia growth factor (VGF) genes, restricting robust viral propagation to tumor tissue. Sequential modifications have added imaging or immunostimulatory modules, such as vvDD-GFP for tracking and vvDD-IL-2 for local immune activation within the tumor microenvironment (85, 91, 92). This stepwise strategy reflects a rational approach to balancing safety, efficacy, and translational relevance. The detailed engineering process of the vvDD series is summarized in **Supplementary Figure S2**.

Collectively, studies across orthotopic, metastatic, and pleural dissemination models—summarized in **Table 2**—demonstrate how these VV platforms perform in diverse pathological settings. The data provide insight into the mechanistic interplay between viral backbones, tumor microenvironment modulation, and context-dependent immune responses, emphasizing the translational potential of VV-based therapies in lung cancer.

**Table 2:** Summary of genetically modified vaccinia virus strains evaluated in lung cancer *in vivo* animal models.

Virus name	Genetic modifications	Model	Antitumor efficacy	PMID
VVΔTKΔN1L-IL12	ListerVVL15 ΔTK,ΔN1L,+IL-12^a^	LLC (C57BL/6, subcutaneous)	NK cell expansion TME inflammation ↑ (IL-1α/β, G-CSF, CXCL1, MIP-1α)^c^ neutrophil recruitment & reprogramming	32217766
VVL-m12	ListerΔTK/ΔN1L; IL-12 knock-in (STC-regulated)^b^	CMT64,LLC	Tcm-mediated long-term immunity TME remodeling:↑CD8^+^,↓Treg,↑DC/M1 Anti-angiogenic effect (↓CD31^+^ vessels)	39840061
GLV-1h68	LIVP,ΔF14.5L/J2R/A56R,+RUC-GFP,+LacZ,+GusA	PC14PE6-RFP	Proinflammatory cytokine upregulation (MCP-1/5, TNF-α)	25030093
vB7/β/IL-12	+B7-1, +IL-12(p35/p40), +lacZ, +gpt	CT26.CL25	95% reduction in lung metastases superior efficacy vs. single-antigen vectors	9862625
VVL-GL21	ΔTK,ΔA49, +GM-CSF, +IL-21	CMT64	CD8^+^/DC/M1↑;TME reprogramming; long-term immunity	39830516
vvDD-mIL2	ΔTK,ΔVGF,+membrane-bound IL-2	bilateral LLC	mIL-2–induced TME activation; ↑ systemic immunity; limited effect on distant tumors	32405533
VV.CXCL11	ΔTK, ΔVGF, +luc, +murine CXCL11	TC1	TME reprogramming; Ag-specific T cells ↑	29399394
VV-IL-2	ΔTK,+mIL-2(GPI-anchored)	LLC Pleural dissemination model	Suppressed pleural metastases;↑ CD8^+^ T cells & TCR diversity; i.pl. > systemic	33485667
vvDD-IL-9	WR.ΔTK, ΔVGF,+murine IL-9,+YFP(tk locus)	LLC	Tumor/metastasis control via IL-9–mediated CCR6^+^ CD8^+^ T cell infiltration	38473379
vvDD-IL-2-RG	WR.ΔTK, ΔVGF, +IL-2(rigid-linker +GPI anchor), +YFP	LLC	Local IL-2–mediated CD8^+^ T cell activation; ↓ systemic toxicity	30410056
WR.TK-HPGD^+^	WR.ΔTK,+mHPGD(GPI-anchored)	LLC	↓MDSC, ↑CD8^+^ T cell, NK cell expansion, Elevated TME inflammation (IL-1α/β, G-CSF, CXCL1, MIP-1α)	27374223
EphA2-TEA-VV	WR.VSC20(ΔTK,ΔVGF),+EphA2-TEA(scFv×2)	A549 subcutaneous xenograft	↓Lung metastasis burden ↑Bystander killing ↑Systemic antitumor immunity	24135899
TG6050	Copenhagen.ΔTK/RR/M2L,+hu-scIL-12&hu-anti-CTLA-4(mAb)	LLC	↓Tumor growth(LLC et al.) ↗Survival,CD8^+^ T cell infiltration,M1/M2 ↑macrophage ratio, ↓Treg accumulation	11284822
VV.mIFNβ	WR. ΔB18R, ΔTK, +Luc & mIFNβ	TC-1	↑Tumor regression, Direct oncolysis: ↑Viral replication & sustained IFNβ expression	22008913
VV.mIFNβ	WR. ΔB18R, ΔTK, +Luc & mIFNβ	LKRM2	↑CD8^+^ T cell response, Immune priming: ↑T-cell activation; Metastasis control (limited viral rep.) Adaptive immunity activation	22008913
GLV-1h68	LIVP,ΔF14.5L/J2R/A56R,+RUC-GFP,+LacZ,+GusA	PC14PE6-RFP	↓ tumor & effusion; VEGF↓; TCCBV clearance; invasion↓	23635329
GLV-1h108	GLV-1h68 + glaf-1 (inserted at J2R locus)	PC14PE6-RFP	↓ tumor & effusion; anti-VEGF–driven antiangiogenesis	23635329
GLV-1h210	GLV-1h68-derived,TK^-^::hEPO(under P7.5E promoter)	A549 subcutaneous	Tumor suppression & anemia improvement via hEPO; ↑ viral spread	23765443
vvDD	WR.ΔTK,ΔVGF	Urethane model; LAP0297,MAD109	Efficacious in refractory lung cancer models, Intravenous/intratumoral administration effective, Increased tumor cell apoptosis, No significant apoptosis in normal lung tissue	32461344

^a^TK,N1L deletion and IL-12 insertion strain.

^b^IL-12 knock-in under STC regulation.

^c^↑, increase or enhancement; ↓, decrease or reduction; ↗, increase (used for survival or functional readouts).

TK, thymidine kinase; Δ, deletion; +, insertion; GFP, green fluorescent protein; STC, synthetic transcriptional control (regulatable promoter system); LacZ, β-galactosidase; hNIS, human sodium iodide symporter; RUC, Renilla luciferase; GusA, β-glucuronidase; IL-12, interleukin-12; GM-CSF, granulocyte-macrophage colony-stimulating factor; IL-21, interleukin-21; IL-2, interleukin-2; IL-9, interleukin-9; mIL-2, murine IL-2; CXCL11, chemokine ligand 11; hEPO, human erythropoietin; P7.5E, P7.5 early promoter; MOI, multiplicity of infection; LC3-I/II, forms of microtubule-associated protein 1A/1B-light chain 3; CD4^+^/CD8^+^ T cell, CD4-positive/CD8-positive T lymphocytes; CCR6^+^, C-C chemokine receptor 6 positive; Tcm, central memory T cells; DC, dendritic cell; M1/M2, macrophage polarization states; Treg, regulatory T cell; ASC, apoptosis-associated speck-like protein containing a CARD; TME, tumor microenvironment; VEGF, vascular endothelial growth factor; i.pl., intrapleural; NK, natural killer cell; LAP0297, murine lung adenocarcinoma line; C57BL/6, mouse strain; LLC, Lewis lung carcinoma; CMT64, murine lung carcinoma; PC14PE6-RFP, human lung adenocarcinoma xenograft; A549, human lung adenocarcinoma cell line; TC-1, murine lung carcinoma; LKRM2, murine lung carcinoma; CT26.CL25, murine colon carcinoma.

### 4.3 Combination strategies and delivery optimization: enhancing efficacy and expanding indications

Combination and delivery strategies in lung cancer models differ conceptually between replication-competent oncolytic vaccinia virus (VV) constructs and non-replicating Modified Vaccinia Ankara (MVA) platforms. Replication-competent VVs rely on intratumoral amplification, direct oncolysis, and local immune remodeling, whereas MVA vectors function primarily as vaccine platforms designed for systemic immune priming without productive tumor replication. Accordingly, preclinical combination studies involving these systems should be interpreted within their distinct therapeutic frameworks. Unless otherwise specified, lung cancer–specific murine models (e.g., LLC, CMT64, TC-1, urethane-induced tumors) are emphasized. Findings derived from non–lung primary tumors or metastatic surrogate models are explicitly indicated.

#### 4.3.1 Combination strategies using replication-competent VV

Combining replication-competent oncolytic VV construct with immune checkpoint inhibitors (ICIs) has consistently demonstrated synergistic effects in lung cancer models. In Lewis lung carcinoma models, pretreatment with VVL-m12 followed by PD-1 blockade led to complete tumor regression in most mice and established durable immune memory. Similarly, a VV vector encoding soluble murine PD-1 (VV-msPD1) alone demonstrated potent localized immunomodulatory effects (25).

Dual checkpoint inhibition further strengthened therapeutic outcomes. In urethane-induced tumors, PD-1/TIM-3 blockade combined with vvDD prevented compensatory immune escape (85, 93, 94). CTLA-4 blockade enhanced the efficacy of cytokine-armed replication-competent vectors such as vvDD-IL-9 in metastatic settings. Beyond checkpoint inhibition, replication-competent VV platforms have shown synergy with conventional and molecularly targeted therapies. Cyclophosphamide augmented intratumoral spread and efficacy of GLV-1h68 (87). In a melanoma lung metastasis model (B16F10-LacZ), epigenetic modulation using the HDAC inhibitor trichostatin A enhanced viral replication by attenuating tumor-intrinsic antiviral responses (54).Additionally, VV constructs designed to remodel the tumor microenvironment, like VV.CXCL11, showed improved outcomes when combined with adoptive cell therapies, including CAR-T cells (37). Cytokine-armed VVs, such as vvDD-IL-2-RG, paired with ICIs or co-stimulatory agonists (e.g., anti-4-1BB), effectively potentiated T/NK cell responses and mitigated immunosuppression in high-burden disease (85, 86, 92).

Collectively, these data illustrate that combination strategies built upon replication-competent VV platforms exploit viral amplification and local immune remodeling to achieve synergistic antitumor effects.

#### 4.3.2 Delivery optimization of replication-competent VV

Optimizing delivery strategies is key to maximizing the therapeutic potential of Optimizing delivery routes is critical for maximizing the therapeutic potential of replication-competent VV constructs, whose efficacy depends on productive tumor infection and effective immune engagement. Systemic administration is particularly relevant for lung cancer, given its frequent metastatic dissemination and pronounced intra-tumoral heterogeneity.

While intratumoral injections can convert tumors into *in situ* vaccination sites, their effects are largely confined to the injected lesion, potentially limiting control over distant micrometastases. Intravenous delivery, as explored with platforms such as VV.mIFNβ, VVL-GL21, and GLV-1hBEVir, enables viral access to disseminated pulmonary metastases while concurrently stimulating systemic antitumor immunity (80).These strategies highlight the potential of VV as a systemic immunotherapy platform capable of targeting both primary tumors and metastatic lesions.

Local administration strategies remain valuable for regionally confined disease. In pleural dissemination models, intrapleural injection of VV-IL-2 provided superior control of tumor burden and malignant effusion compared with systemic delivery (82), emphasizing the importance of context-dependent route selection. Direct intratumoral injection can also prime systemic immunity, as demonstrated with vvDD-mIL2 combined with TLR9 agonists, which generated immune responses at distant untreated tumors (83). Neoadjuvant approaches offer additional opportunities; preoperative administration of VVΔTKΔN1L prolonged postoperative survival in murine models, with efficacy dependent on NK cell activation (64). Innovative carrier systems have further expanded the toolbox for systemic delivery. In a breast cancer lung metastasis model (AT-3), the BEVir platform delivered VV encoding a bispecific T cell engager (TCE) to pulmonary metastases, enabling localized immune redirection and improved survival (83). Such carriers, combined with rational viral engineering—arming with cytokines, co-stimulatory molecules, or bispecific antibodies—can enhance tumor targeting, overcome microenvironmental immunosuppression, and amplify systemic T cell–mediated antitumor responses (37, 93, 95).

#### 4.3.3 Vaccine-oriented combination approaches using MVA platforms

In contrast to replication-competent VV systems, Modified Vaccinia Ankara (MVA)–based vectors have been evaluated primarily as non-replicating vaccine platforms designed to enhance antigen-specific immunity rather than to mediate direct tumor oncolysis.

In a HER2^+^ colorectal carcinoma lung metastasis model (CT26-HER2), MVA-HER2 vaccination demonstrated improved efficacy in metastatic lung cancer models when combined with CTLA-4 blockade, indicating synergy between antigen-specific priming and checkpoint modulation (75, 93). Similarly, co-administration of MVA vectors with immune adjuvants such as αCD40 enhanced T cell activation and tumor regression (96). In a colorectal carcinoma lung metastasis model (CT26-MUC1), pharmacologic modulation using simvastatin further augmented MVA-induced T cell responses, supporting the concept that host immune conditioning can strengthen vaccine efficacy (97).

These studies underscore that MVA-based approaches operate through systemic immune priming mechanisms distinct from the intratumoral replication paradigm of oncolytic VV. As such, their combination logic centers on amplifying adaptive immune responses rather than enhancing viral propagation.

**Table 3** summarizes key findings from replication-competent VV combination and delivery studies, while vaccine-oriented MVA strategies are presented separately to reflect their distinct mechanistic basis. **Supplementary Table S1** provides a comprehensive overview of viral backbones, genetic modifications, and functional payloads across platforms. While most studies focus on lung cancer–specific models, some combinations were evaluated in lung metastasis models originating from non-pulmonary primary tumors (e.g., colorectal carcinoma, melanoma, or breast cancer). These examples highlight the versatility of the viral platforms, though caution is warranted when extrapolating efficacy to primary lung tumors.

**Table 3:** VV-based combination therapies and delivery strategies.

Virus name	Model	Combination/delivery strategy	Antitumor efficacy	PMID
VVΔTKΔN1L	LLC (C57BL/6, subcutaneous)	Intratumoral injection as neoadjuvant therapy^a^	prolonged postoperative survival, therapeutic efficacy strictly dependent on NK cells, independent of adaptive immunity	32217766
VVL-m12	CMT64,LLC	Sequential VVL-m12 priming + PD-1 axis blockade (anti–PD-1 or VV-msPD1)^b^	>90% CR (no recurrence) VVL-m12 priming + PD-1 blockade → synergistic immunity^g^	39840061
vvDD WR.ΔTK,ΔVGF	Urethane model; LAP0297	Dual blockade of PD-1 and TIM-3;intravenous injection^c^	↓Tumor burden; ↑CD4^+^/CD8^+^ T cell activation; ↑Survival	32461344
GLV-1h68	PC14PE6-RFP	cyclophosphamide (CPA)^d^	↑Virus spread ×2.5,↓VCAM-1/vWF/FG,↓EGF;↓Invasion/Evasio	25030093
vB7/β/IL-12	CT26.CL25	exogenous IL-12 administration^e^	↑Survival + ↑Antitumor T cell response	9862625
TG4010 (MVA-MUC1-IL-2)	CT26-MUC1 lung metastasis	+ anti–PD-1/PD-L1	↑CD8^+^ T cells; +PD-1^+^ exhausted T & Treg; ↓immunosuppression	28925793
VVdd, WR.ΔTK,ΔVGF; TK::GFP	B16F10-LacZ Lung Metastasis Model	Histone deacetylase (HDAC) inhibitors, such as trichostatin A (TSA)	↑replication & intratumoral spread,↑ oncolytic activity; mediated by ↓ tumor-intrinsic innate immunity	21283510
vvDD-mIL2	bilateral LLC	TLR9 agonist CpG	↑ Systemic immunity, ↓ Non-injected tumor, ↑ Survival	32405533
		+ CpG +anti–PD-1 antibody/macrophage^f^ depletion	↓ Immunosuppression in TME, ↑↑ Systemic antitumor effect	32405533
VV.CXCL11	TC1	cancer vaccines or CAR-T cell therapy	exhibited synergistic antitumor effects	29399394
vvDD-IL-9	LLC	CTLA-4 inhibitor	a subset of Complete tumor regression in a subset of mice, Rechallenge protection	38473379
vvDD-IL-2-RG	LLC	anti–PD-1 or anti–PD-L1 antibodies	↓Immunosuppression within TME,↑ Therapeutic efficacy in high-burden tumors	30410056
vvDD	AT-3 lung metastasis model	agonistic anti–4-1BB antibody	↑CD8^+^ T cells,↑NK cells,↑neutrophils,↑IFN-γ signaling, ↓lung metastasis	22315352
MVA-BN-HER2,MVA.ΔNS+HER2^ECD+2×Th	CT26-HER2 lung metastasis model	CTLA-4 blockade	Synergistic efficacy, Enhanced polyfunctional T-cell activation, Prolonged overall survival	26961085
MVA-gp70, MVA.ΔNS + MuLV gp70	LLC-OVA	co-administration of simvastatin	Transient IFN-α/β suppression; enhanced antitumor efficacy	34321273
EphA2-TEA-VV	A549 intravenous model	peripheral blood mononuclear cells	significantly suppressed tumor growth, prolonged survival	24135899
VV.mIFNβ	TC1	Ad.E7 vaccine	significantly boost CD8^+^ T cell infiltration and tumor regression	22008913

^a^Intratumoral neoadjuvant injection of VVΔTKΔN1L prior to surgical resection to assess postoperative survival and NK-cell–dependent efficacy.

^b^Sequential VVL-m12 priming followed by PD-1 axis blockade (anti–PD-1 or VV-msPD1) to evaluate synergistic immunity and >90% complete response in tumor models.

^c^Dual blockade of PD-1 and TIM-3 via intravenous injection in vvDD-treated mice to assess T cell activation and survival benefit.

^d^Cyclophosphamide pre-treatment to enhance viral spread and reduce tumor adhesion/invasion factors.

^e^Exogenous IL-12 administration to enhance antitumor T cell responses and survival.

^f^Co-administration of CpG + anti–PD-1 antibody/macrophage depletion to reduce immunosuppression and boost systemic antitumor immunity.

^g^↑, increase or enhancement; ↓, decrease or reduction; →, lead to.

TK, thymidine kinase; Δ, deletion; GFP, green fluorescent protein; LacZ, β-galactosidase; hNIS, human sodium iodide symporter; RUC, Renilla luciferase; GusA, β-glucuronidase; IL-12, interleukin-12; GM-CSF, granulocyte-macrophage colony-stimulating factor; IL-21, interleukin-21; IL-2, interleukin-2; IL-9, interleukin-9; mIL-2, murine IL-2; CXCL11, chemokine ligand 11; hEPO, human erythropoietin; P7.5E, P7.5 early promoter; MOI, multiplicity of infection; LC3-I/II, forms of microtubule-associated protein 1A/1B-light chain 3; CD4^+^/CD8^+^ T cell, CD4-positive/CD8-positive T lymphocytes; CCR6^+^, C-C chemokine receptor 6 positive; Tcm, central memory T cells; DC, dendritic cell; M1/M2, macrophage polarization states; Treg, regulatory T cell; ASC, apoptosis-associated speck-like protein containing a CARD; TME, tumor microenvironment; VEGF, vascular endothelial growth factor; i.pl., intrapleural; NK, natural killer cell; HDAC, histone deacetylase; CPA, cyclophosphamide; STC, synthetic transcriptional control (regulatable promoter system); B16F10-LacZ, murine melanoma lung metastasis model; LLC, Lewis lung carcinoma; C57BL/6, mouse strain; CMT64, murine lung carcinoma; PC14PE6-RFP, human lung adenocarcinoma xenograft; A549, human lung adenocarcinoma cell line; TC-1, murine lung carcinoma; LKRM2, murine lung carcinoma; CT26.CL25, murine colon carcinoma; CT26-MUC1, murine lung metastasis model expressing MUC1; LLC-OVA, LLC expressing ovalbumin; AT-3, murine mammary adenocarcinoma; MVA-BN-HER2, modified vaccinia Ankara expressing HER2; MuLV gp70, murine leukemia virus gp70 antigen; Ad.E7, adenoviral E7 vaccine.

## 5 Early clinical insights into vaccinia virus–based immunotherapy for lung malignancies

Clinical development of vaccinia virus (VV)–based immunotherapies in lung malignancies has progressed through two principal platforms: replication-competent oncolytic viruses and non-replicating Modified Vaccinia Ankara (MVA)–based vaccines. While replicating VVs combine direct oncolysis with immune activation, MVA-based constructs primarily function as immunogenic vectors designed to enhance tumor-specific T-cell responses. Distinguishing these strategies is important for accurate interpretation of clinical outcomes (98). To date, most studies have been early-phase trials enrolling heterogeneous solid tumor populations, with lung cancer frequently represented as a subset rather than a predefined primary cohort. As a result, available data largely emphasize safety, viral biodistribution, and immunologic activity, whereas definitive conclusions regarding clinical efficacy in non-small cell lung cancer (NSCLC) remain limited (39). Overall, VV-based platforms have demonstrated acceptable tolerability and measurable biological activity, including evidence of tumor infection and immune activation. However, objective tumor responses in lung cancer cohorts have been infrequent, and most trials were not powered to establish durable antitumor benefit. The following sections organize clinical findings by platform type—MVA-based vaccines and replication-competent oncolytic VVs—and present outcomes hierarchically, distinguishing safety, immune activation, and objective tumor responses.

### 5.1 MVA-based therapeutic vaccines in NSCLC

Modified Vaccinia Ankara (MVA)–based platforms represent a non-replicating vaccinia strategy designed primarily to enhance tumor-specific immune responses rather than to induce direct oncolysis. In non-small cell lung cancer (NSCLC), clinical investigation has focused mainly on antigen-directed therapeutic vaccines, among which TG4010 is the most extensively studied.

#### 5.1.1 TG4010 (MVA-MUC1-IL2)

TG4010 encodes the tumor-associated antigen MUC1 together with interleukin-2 (IL-2), aiming to promote antigen-specific T-cell priming and support NK-cell activity. In the randomized phase IIb/III TIME trial (NCT01383148), TG4010 was added to first-line platinum-based chemotherapy in advanced NSCLC. TG4010 demonstrated acceptable tolerability, with predominantly mild injection-site reactions and transient systemic symptoms, supporting its feasibility in combination regimens. No lung-specific adverse events were reported, but systematic assessment of pulmonary safety was not conducted in this trial, limiting conclusions regarding VV-induced pulmonary toxicity in NSCLC patients. Immunologically, treatment was associated with evidence of vaccine-induced immune activation. Exploratory analyses suggested that a predefined immune-related biomarker (TrPAL) may correlate with differential clinical outcomes, underscoring the potential importance of patient selection. Clinically, the addition of TG4010 was associated with statistically significant differences in progression-free survival and objective response rate in the overall study population. However, the magnitude of benefit was modest and heterogeneous across subgroups, and the study design does not allow clear attribution of long-term benefit specifically to the vaccine component. Thus, TG4010 demonstrates immunogenicity and manageable safety, while its definitive contribution to durable antitumor efficacy in NSCLC remains to be established (99).

#### 5.1.2 Other MVA-based vaccine approaches

Other MVA-based constructs targeting tumor-associated antigens have been evaluated in early-phase studies that included lung cancer subsets. These platforms have generally shown favorable safety profiles and measurable immunogenicity, including antigen-specific T-cell responses. However, lung cancer–specific efficacy data remain limited, as most studies enrolled heterogeneous solid tumor populations and were not powered for definitive outcome assessment. Objective tumor regressions attributable to MVA vaccination alone have been uncommon. Overall, current evidence positions MVA-based therapeutic vaccines in NSCLC primarily as immunogenic platforms with acceptable safety, whose clinical impact likely depends on biomarker-guided selection and rational combination with contemporary immunotherapies rather than on standalone cytotoxic effects.

### 5.2 Replicating oncolytic vaccinia virus platforms

Replication-competent vaccinia viruses preferentially replicate in tumor cells, combining direct oncolysis with immune activation. Clinical experience in lung malignancies is largely limited to early-phase trials enrolling heterogeneous solid tumor populations, with few lung-specific cohorts.

#### 5.2.1 Intravenous administration (GL-ONC1, JX-594, ASP9801)

Systemic delivery of GL-ONC1, JX-594 (Pexa-Vec), and ASP9801 has generally been well tolerated, with mild flu-like symptoms and rare dose-limiting toxicities (100). Viral DNA has been transiently detected in plasma, and tumor biopsies confirm intratumoral viral presence in select patients, supporting systemic tumor targeting (101, 102).

Immune monitoring has shown cytokine induction and increased tumor-infiltrating lymphocytes in some cases, indicating immune activation. Objective tumor responses, however, have been uncommon, mostly limited to stable disease, and lung cancer–specific efficacy remains unestablished due to small sample sizes and heterogeneous populations.

#### 5.2.2 Locoregional administration (intrapleural GL-ONC1)

Intrapleural GL-ONC1 delivery was feasible and tolerated, with mild, transient systemic symptoms(NCT01766739). Viral replication was confirmed in pleural fluid, and increased CD8^+^ T-cell and NK-cell infiltration indicated local immune activation. Clinical impact in NSCLC patients was limited, with early progression observed, suggesting that locoregional replication alone is insufficient for durable control.

### 5.3 Cross-platform clinical signals and current limitations

Early-phase studies of both MVA-based vaccines and replication-competent oncolytic vaccinia viruses indicate generally acceptable safety and measurable immune activation. Adverse events are mostly mild, such as transient flu-like symptoms or injection-site reactions, with serious toxicities uncommon.

Immune effects include cytokine induction, increased tumor-infiltrating lymphocytes, and antigen-specific T-cell responses. Tumor trafficking has been confirmed for replication-competent platforms. Objective tumor responses remain limited, typically stable disease, and durable regressions are rare. Most trials enrolled heterogeneous solid tumor populations, with lung cancer often a small subset, limiting lung-specific conclusions.

Overall, VV-based immunotherapies are biologically active and safe, but current data do not establish definitive efficacy in NSCLC. Small sample sizes and limited lung-specific reporting highlight the need for dedicated studies.

### 5.4 Pulmonary immune toxicity and lung-specific safety considerations

Pulmonary safety is a critical consideration for VV-based therapies, especially in lung cancer or thoracic administration. Clinical evidence remains limited, but early-phase studies suggest lung-related adverse events are uncommon and generally mild.

Systemic administration of replication-competent VVs (GL-ONC1, JX-594, ASP9801) rarely caused pulmonary toxicity. Observed effects were mostly transient flu-like symptoms; no severe pneumonitis or respiratory compromise was reported. Viral trafficking to pulmonary lesions occurred without clear evidence of clinically significant lung injury.

Locoregional delivery (intrapleural GL-ONC1) exposes lung tissue to higher viral load. Mild, transient systemic symptoms were noted, and pleural biopsies confirmed immune activation (CD8^+^ T-cell and NK-cell infiltration). Despite local viral replication, NSCLC patients progressed rapidly, indicating that intrathoracic administration alone does not provide meaningful tumor control.

Mechanistically, type I interferon and cytokine induction may mediate mild, reversible pulmonary inflammation. No serious or irreversible events were observed, but the small cohort sizes and heterogeneous populations limit firm conclusions. Systematic monitoring of lung function, imaging, and inflammatory markers is warranted in future studies.

In summary, early data indicate VV therapies are feasible and largely safe for pulmonary exposure, but lung-specific risks are incompletely characterized, and efficacy in NSCLC remains unproven.

## 6 Integrated mechanisms of vaccinia virus–mediated anti-tumor immunity in lung cancer

Vaccinia virus (VV) exerts antitumor effects in lung cancer through a combination of direct cytolysis and immune modulation (103). Beyond lysing tumor cells, VV induces immunogenic cell death (ICD), activates tumor-specific adaptive immunity, and remodels the immunosuppressive tumor microenvironment (TME), collectively generating potent local and systemic antitumor responses in preclinical lung cancer models (70).

**Figure 3:**
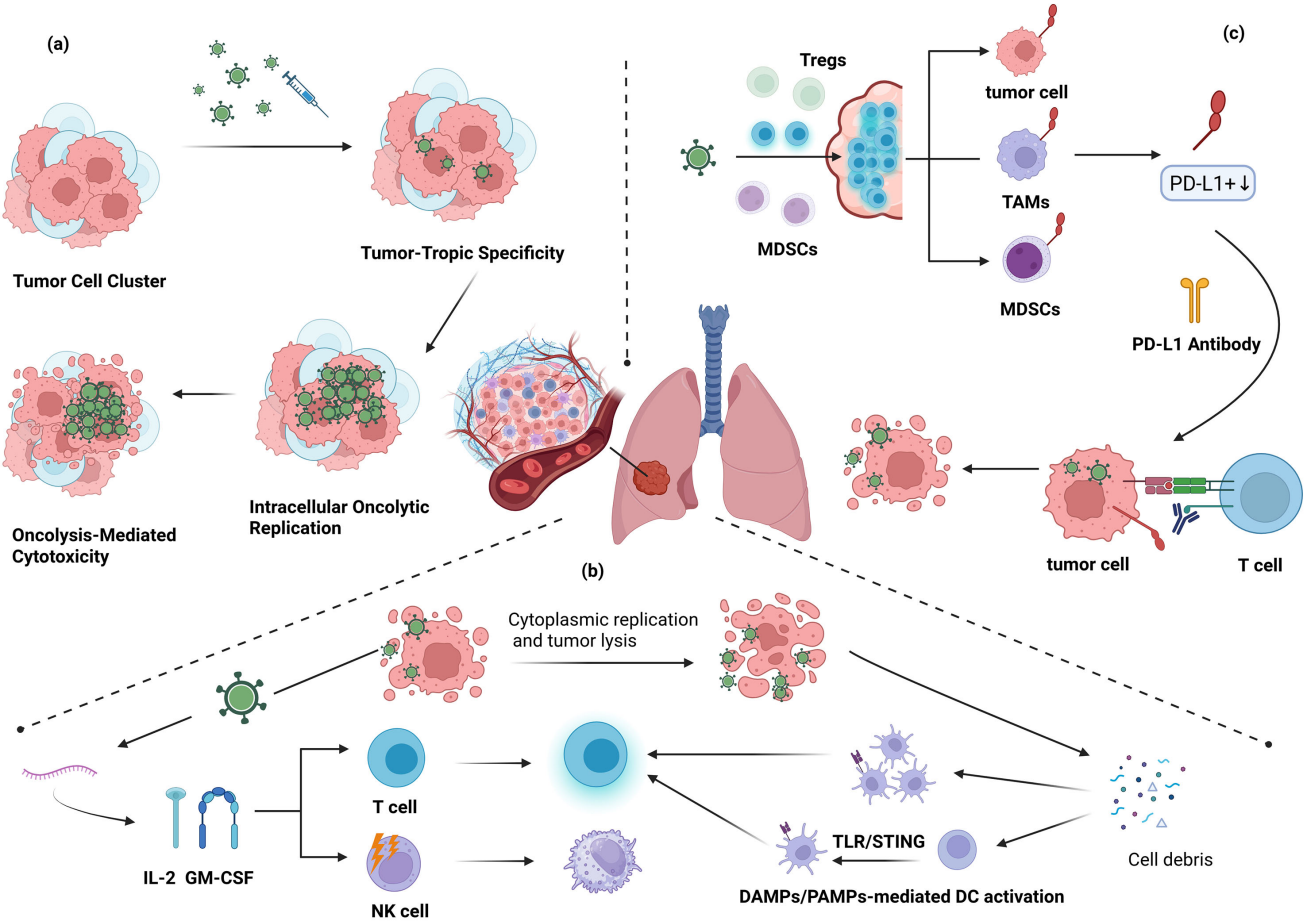
Local antitumor effects of VV, vaccinia virus in lung cancer therapy. **(a)** Cytoplasmic replication and direct lysis of tumor cells. **(b)** Lysis-induced release of PAMPs/DAMPs activates DCs, dendritic cells and enhances antigen presentation; in parallel, engineered expression of cytokines (e.g., IL-2, GM-CSF) recruits and activates T and NK cells. **(c)** VV infection downregulates PD-L1 expression or synergizes with PD-1 blockade to overcome local immunosuppression, leading to increased CD8^+^ T-cell infiltration and reduced accumulation of MDSCs and Tregs.

**Figure 4:**
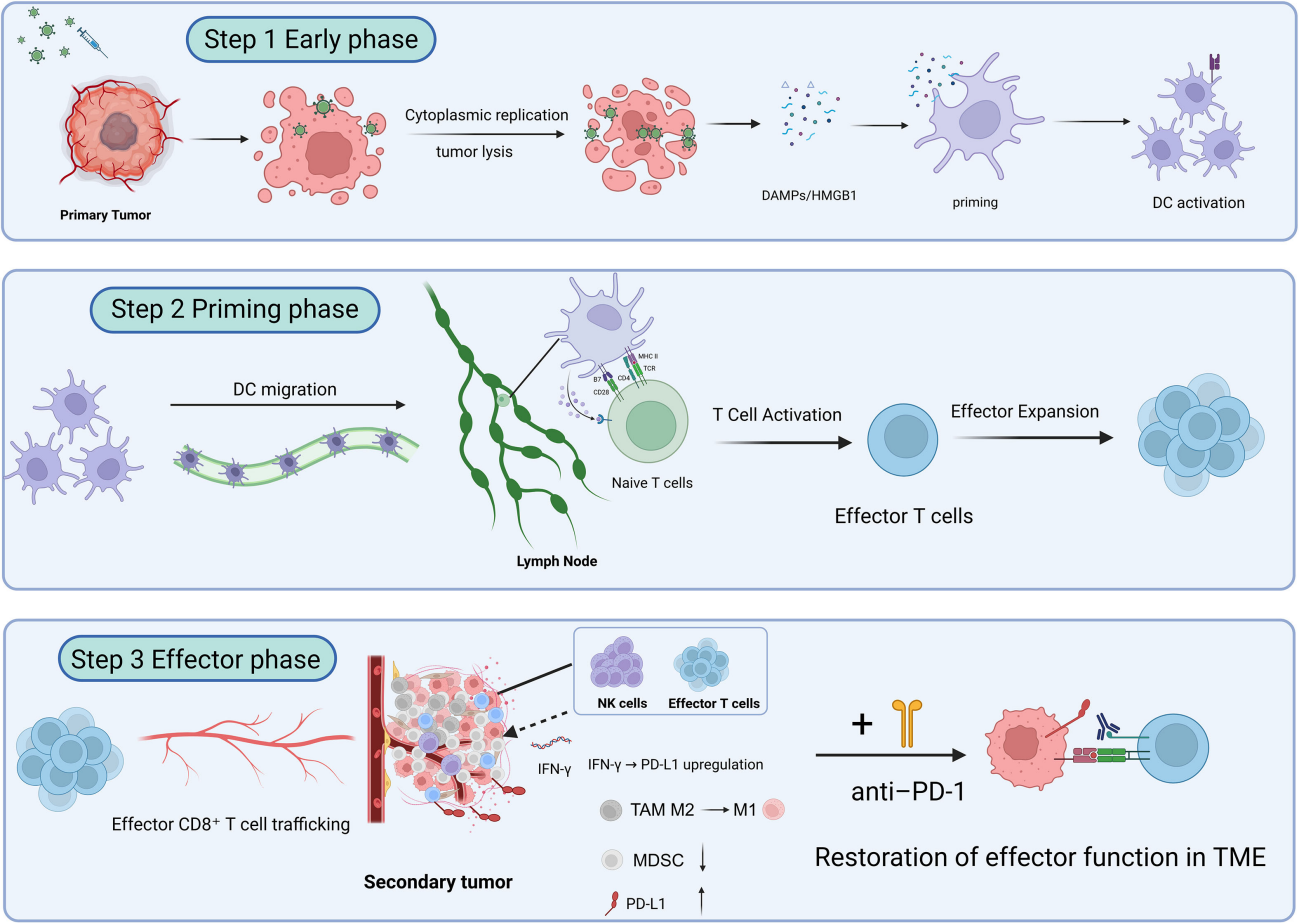
Systemic immune activation cascade triggered by VV, vaccinia virus -mediated lysis of lung cancer cells. VV infection induces tumor cell lysis and antigen release at the tumor site, promoting dendritic cell activation. Antigen-loaded dendritic cells migrate to draining lymph nodes to prime tumor-specific CD8^+^ T cells. Activated effector T cells then re-enter systemic circulation, traffic back to tumor sites, and mediate cytotoxic tumor killing, accompanied by remodeling of the tumor microenvironment. Immune checkpoint blockade may further enhance this response.

As illustrated in **Figures 3**, **4**, VV-mediated immunity follows a coordinated sequence: oncolysis and ICD release tumor antigens and danger signals, which stimulate dendritic cell maturation and prime tumor-specific T-cells, establishing systemic immune surveillance. Genetically engineered VVs can enhance these processes and promote a pro-inflammatory, immune-permissive TME. It should be noted, however, that the magnitude and nature of these responses are context-dependent, varying with viral strain, host genetic background, and tumor model, and may not be fully generalizable beyond the preclinical systems studied.

These mechanisms can be summarized in five interconnected modules:

Direct Oncolysis and ICD: VV replication lyses cancer cells while triggering ICD, engaging both innate and adaptive immunity.

Innate Immune Priming via STING–Batf3: Viral sensing activates STING in antigen-presenting cells, driving Batf3-dependent dendritic cell cross-priming of T-cells.

Enhanced Antigen Presentation and Costimulation: Engineered VVs can deliver tumor antigens or co-stimulatory signals to broaden and strengthen systemic T-cell responses.

Tumor Microenvironment Remodeling: VV reduces immunosuppressive cell populations and fosters a T-cell-inflamed, pro-inflammatory TME.

Rational Combination Strategies: VV-induced immunity provides a foundation for synergistic therapies with immune checkpoint inhibitors and other immunotherapies.

The following sections detail each module, exploring molecular mechanisms, immunological consequences in lung cancer, and bioengineering strategies to maximize efficacy in preclinical and clinical settings.

### 6.1 Direct oncolysis and the induction of immunogenic cell death

The antitumor efficacy of vaccinia virus (VV) is primarily driven by its preferential infection and lysis of tumor cells. Beyond direct cytolysis, VV induces immunogenic cell death (ICD), linking local tumor destruction to systemic immune activation (29, 104). In this process, dying tumor cells release damage-associated molecular patterns and tumor antigens that promote dendritic cell maturation and cross-presentation, effectively priming CD8^+^ T cell responses (105, 106). Rational viral engineering can further amplify ICD. The vvDD strain, deficient in thymidine kinase (TK) and vaccinia growth factor (VGF), exhibits enhanced tumor selectivity and robust ICD induction through augmented lytic activity and local immune stimulation. Similarly, engineered viruses such as GLV-1h209 improve dendritic cell activation and facilitate T-cell infiltration into the tumor (26, 107) (**Figure 3**).In lung cancer models—including Lewis lung carcinoma and A549 cells—vvDD infection elevates ICD markers, promoting efficient dendritic cell uptake of tumor antigens and subsequent T-cell priming (108). These observations underscore the potential of optimized VV strains to act as *in situ* vaccines, converting poorly immunogenic tumors into immune-responsive environments and enabling durable, systemic antitumor responses (53, 90).

In summary, ICD functions as a pivotal mediator linking VV-mediated oncolysis to adaptive immunity, highlighting its relevance for the design of next-generation viral immunotherapies and combination strategies (109). It is important to note that these observations are derived from preclinical lung cancer models, and the extent to which ICD contributes to systemic immunity may vary across tumor types and in clinical settings.

### 6.2 Context-dependent activation of the STING–Batf3 axis in adaptive immune priming

The generation of durable, tumor-specific CD8^+^ T cell responses is critically dependent on Batf3^+^ conventional type 1 dendritic cells (cDC1s), which specialize in cross-presenting tumor-associated antigens to naïve T cells (110). In preclinical models utilizing poxviral vectors, activation of cytosolic DNA sensing pathways has been implicated in facilitating this cDC1-dependent immune priming (55).

Compelling mechanistic evidence has been derived from studies employing intratumoral administration of inactivated Modified vaccinia Ankara (iMVA) in transplantable tumor models. In C57BL/6 mice bearing B16-F10 melanoma or MC38 colon carcinoma, iMVA induced robust type I interferon production through cGAS–STING activation in dendritic cells and tumor cells (39). In these systems, antitumor efficacy was abrogated in STING-deficient or Batf3-deficient mice, and CD8^+^ T cell depletion eliminated therapeutic benefit, demonstrating a strong dependence on STING signaling and Batf3^+^ cDC1s under these experimental conditions. Notably, intratumoral iMVA also generated systemic antitumor immunity in bilateral tumor models, supporting its capacity to initiate adaptive immune priming beyond the injected lesion (19, 20).

However, several considerations temper generalization of this mechanism. First, these findings were established in transplantable murine tumor models rather than genetically engineered lung cancer models. Second, the viral platform employed was non-replicative iMVA, which differs biologically from replicative vaccinia virus strains in terms of cytoplasmic DNA abundance and innate sensing dynamics. Third, host genetic background and immune competence may influence the relative requirement for STING signaling in shaping dendritic cell responses (111). In lung cancer–relevant systems, including studies using recombinant MVA-based vaccines combined with PD-1/PD-L1 blockade, enhanced CD8^+^ T cell infiltration and therapeutic synergy have been observed; however, direct interrogation of the STING–Batf3 axis has not consistently been performed in these models (43, 111, 112) (**Figure 4**).

Thus, while STING activation represents a prominent mechanism in certain poxvirus-based immunotherapy platforms, current evidence supports a context-dependent rather than universally required role for the STING–Batf3 axis in vaccinia-mediated adaptive immune priming (39, 97).

While STING–Batf3 activation represents a potentially important mechanism, direct evidence in lung cancer–specific models remains limited, and extrapolation to human disease should be made cautiously.

### 6.3 Antigen-encoding and costimulatory strategies of VV to promote systemic immunity

A key goal in cancer immunotherapy is eliciting a durable, tumor-specific CD8^+^ T cell response. Vaccinia virus (VV) provides a naturally inflammatory environment, but its value as a vaccine platform is realized through genetic modification to deliver defined tumor antigens along with costimulatory signals (113), potentially overcoming immune ignorance or tolerance in tumors such as NSCLC.

VV’s large genome (~192 kb) allows stable insertion of full-length antigens and immunomodulatory genes (114). GLV-1h68-derived vectors (PC14PE6-RFP, A549 xenografts) and vvDD platforms (bilateral LLC, metastatic models) have demonstrated tumor control, immune activation, and reporter-based tracking in preclinical studies (115). Replication-incompetent platforms such as VIReST enhanced CD8^+^ T cell priming, intratumoral infiltration, and survival in KP lung cancer models (116).

Costimulatory strategies further improve responses. TRICOM-expressing VVs (B7-1/CD80, ICAM-1, LFA-3) and multi-gene constructs—TG6050 (IL-12 + anti-CTLA-4), vB7/β/IL-12, VVL-GL21 (GM-CSF + IL-21)—in LLC and CMT64 models increased CD8^+^ T cell infiltration, shifted macrophages toward M1, reduced Tregs, remodeled the TME, and prolonged survival (116). VVL-m12 promoted central memory T cell formation, suggesting potential for durable immunity.

In summary, in multiple preclinical lung cancer models, engineering VV to encode tumor antigens and costimulatory signals has enhanced systemic, antigen-specific immunity, supporting its development as a versatile, modular platform for personalized immunotherapy. Although these strategies have demonstrated enhanced antigen-specific immunity in multiple preclinical lung cancer models, the translational relevance may be influenced by the immunogenicity of individual tumors and the choice of viral backbone (38).

### 6.4 Tumor microenvironment remodeling and inflammation-driven immune infiltration

Non-small cell lung cancer (NSCLC) is frequently characterized by a profoundly immunosuppressive tumor microenvironment (TME), with limited tumor-infiltrating lymphocytes (TILs), abundant myeloid-derived suppressor cells (MDSCs), M2-polarized tumor-associated macrophages (TAMs), and stromal barriers that collectively contribute to therapeutic resistance (26, 107, 117, 118) (**Figure 4**). In lung cancer models, VV infection has been shown to initiate robust local inflammation that reshapes this suppressive milieu. Specifically, in Lewis lung carcinoma and other NSCLC models, VV replication induces type I interferons and pro-inflammatory cytokines, promoting recruitment of NK cells, dendritic cells, and monocytes. This inflammatory reprogramming enhances CD8^+^ T-cell infiltration and sensitizes tumors to subsequent checkpoint blockade (31, 119). Moreover, in lung cancer settings employing GLV-1h68 and vvDD, VV treatment has been associated with TAM repolarization from an M2 to M1 phenotype and a reduction in MDSC abundance, thereby alleviating local immune suppression. These findings underscore that TME remodeling is not merely a generalized OV phenomenon but has been specifically demonstrated in lung cancer preclinical systems (39, 120). Engineering strategies further potentiate these effects. In lung cancer models, VV strains expressing IL-12, GM-CSF, or 15-hydroxyprostaglandin dehydrogenase (15-PGDH) enhance CD8^+^ T-cell infiltration and IFN-γ production, improving synergy with immunotherapies (39, 84). Advanced platforms such as VIReST, evaluated in KP lung cancer models, combine antigen delivery with inflammatory conditioning and have demonstrated significant enhancement of T-cell recruitment and TME restructuring (116, 121, 122). Collectively, evidence from lung cancer–specific preclinical models indicates that VV-mediated TME remodeling overcomes local immune suppression and establishes an immune-permissive niche that supports durable antitumor immunity. Importantly, the degree of TME remodeling and immune infiltration induced by VV may differ across tumor models and host immune contexts, underscoring the need for careful interpretation when translating these findings to human lung cancers (57).

### 6.5 Rationale for combination strategies: VV-induced upregulation of immune checkpoints

Vaccinia virus–based platforms can induce substantial inflammatory responses in preclinical tumor models, but the magnitude and therapeutic implications of this activation vary with viral strain and host immune context. In immunocompetent murine systems, inflammatory cytokines such as IFN-γ have been associated with upregulation of inhibitory molecules including PD-L1 and IDO1 on tumor cells and antigen-presenting cells (39, 90) (**Figure 4**). Concurrently, tumor-infiltrating CD8^+^ T cells frequently upregulate PD-1 following viral priming—a phenotype consistent with activation-associated checkpoint expression, although the extent of functional exhaustion may differ across models (67, 123).

Evidence for therapeutic exploitation of this response derives primarily from transplantable lung tumor models in immune-competent mice. In C57BL/6 mice bearing syngeneic Lewis lung carcinoma (LLC), replicative vaccinia strains such as vvDD and GLV-1h151 were associated with increased CD8^+^ T cell infiltration and concomitant PD-L1 upregulation within the tumor microenvironment (39, 41, 91).

Similarly, recombinant Modified vaccinia Ankara–based vaccines evaluated in transplantable lung tumor systems augmented T cell recruitment and rendered tumors sensitive to subsequent PD-1/PD-L1 blockade. In these defined experimental settings, sequential checkpoint inhibitor administration partially restored T cell effector function and improved tumor control compared with monotherapy (94, 124).

Beyond PD-1/PD-L1, evidence supporting dual PD-1/CTLA-4 blockade or targeting of additional inhibitory receptors such as LAG-3 and TIM-3 remains model-specific and has not been uniformly validated across lung cancer systems. Moreover, these observations have not yet been extended to genetically engineered lung cancer models or humanized systems, which may more faithfully recapitulate the complexity of the tumor microenvironment (124). Collectively, available data suggest that vaccinia-induced checkpoint upregulation represents a context-dependent adaptive response that may be therapeutically exploitable. However, optimal sequencing, viral platform selection, and immune checkpoint targets likely depend on tumor model, host genetic background, and the replicative capacity of the viral vector—variables that warrant systematic evaluation in future studies. Overall, while these preclinical observations support the potential of VV-based combinations with checkpoint blockade, optimal sequencing, viral platform choice, and target selection will likely require systematic evaluation in clinically relevant models before extrapolation to patients.

## 7 Challenges and future directions

### 7.1 Translational challenges and innovative strategies in VV-based lung cancer immunotherapy

Vaccinia virus (VV) holds promise in lung cancer therapy, serving both as a direct oncolytic agent and a platform for tumor-targeted immunization. Despite encouraging preclinical and early clinical results, several obstacles limit its broad clinical application. Effective translation will require not only the optimization of viral engineering and delivery strategies but also innovative approaches to modulate the tumor-immune interface. The following sections highlight major translational challenges and emerging strategies aimed at fully realizing the potential of VV-based therapies in lung cancer.

#### 7.1.1 Enhancing VV-induced long-term antitumor immunity

A central challenge in vaccinia virus (VV) therapy for lung cancer is the difficulty in generating durable, tumor-specific CD8^+^ T cell responses. Preclinical models often show early effector peaks followed by waning activity, reflecting tumor antigen heterogeneity and immunosuppressive microenvironments (63, 125). Multi-gene constructs combining IL-12 with costimulatory signals or checkpoint blockade (TG6050, vB7/β/IL-12, VVL-GL21) further enhanced systemic immunity and remodeled “cold” tumors into more immunogenic microenvironments (43). Targeted T cell recruitment (VV.CXCL11, EphA2-TEA-VV) and localized cytokine delivery (vvDD-IL-2 variants) demonstrated potential for eliciting systemic responses, including at distant tumor sites (126). Despite these advances, limitations remain. Durable memory formation has been primarily demonstrated in murine models; translation to human lung tumors is uncertain. Tumor heterogeneity, immune suppression, and differences in viral tropism pose challenges for predictable, long-term efficacy. In addition, tumor plasticity can dynamically modify neoantigen expression, HLA presentation, and immune checkpoint levels, potentially limiting the generation of durable, systemic CD8^+^ T cell responses (74, 127).This dynamic behavior reinforces the need for multi-antigen or adaptable VV strategies capable of maintaining effective immune surveillance despite evolving tumor phenotypes. Moreover, safety concerns with systemic cytokine expression and multi-gene constructs require careful evaluation. Looking forward, future efforts should integrate rational platform engineering with patient-specific antigen selection, combine VV with checkpoint inhibitors or immune agonists, and leverage longitudinal immune monitoring to guide adaptive strategies. Such approaches may enable VV to evolve from a transient oncolytic trigger into a modular platform capable of sustained systemic antitumor immunity in lung cancer.

#### 7.1.2 Sustained tumor microenvironment reprogramming

The immunosuppressive microenvironment in lung cancer—dominated by tumor-associated macrophages (TAMs), myeloid-derived suppressor cells (MDSCs), and regulatory T cells (Tregs)—can rapidly counteract the initial inflammatory response triggered by vaccinia virus (VV), limiting therapeutic benefit (128).

Future strategies should aim to transform VV from a transient inflammatory stimulus into a tool for lasting TME reprogramming. This can be achieved by engineering viruses to degrade inhibitory mediators, such as overexpressing 15-PGDH to reduce prostaglandin E_2_ (PGE_2_) or targeting COX-2 and IDO pathways. Concurrently, VVs can be designed to promote TAM polarization toward an M1-like, antitumor state and to restrict MDSC recruitment or suppressive activity (71, 84, 129).

Validation requires rigorous testing in orthotopic and metastatic lung cancer models, with endpoints including shifts in effector-to-suppressor cell ratios, sustained cytokine changes, and improved spatial organization of immune infiltrates.

By creating a durable, immune-permissive TME, engineered VV therapy can enhance both the magnitude and persistence of antitumor immune responses, laying the foundation for more effective combination strategies (12, 130, 131).

#### 7.1.3 Personalized antigen-encoding strategies in VV therapy

A major hurdle in lung cancer immunotherapy is the limited number of highly immunogenic tumor antigens and the considerable heterogeneity of neoantigen expression across patients and within tumors (10, 132). Conventional vaccines targeting shared tumor-associated antigens (TAAs) often fail to elicit strong, patient-specific T cell responses.

Advancing VV-based therapy requires integrating viral platforms with advanced antigen discovery tools. Techniques such as whole-exome sequencing, transcriptomics, and multi-omic analyses can identify patient-specific, clonally expressed neoantigens with high immunogenic potential (133). These antigens can then be encoded into engineered VV vectors.

Platforms like VIReST (Virus-Infected Reprogrammed Somatic cell Therapy) illustrate this approach. By infecting iPSC-derived, patient-specific tumor cells with replication-incompetent VV strains, VIReST presents a broad and individualized neoantigen repertoire within a highly immunogenic viral context (116, 122). Prime-boost strategies combining VV with other vaccine platforms, such as mRNA or adenoviral vectors, can further expand T cell responses while minimizing the impact of pre-existing anti-vector immunity (134, 135).

Nevertheless, tumor plasticity may result in temporal or spatial variability in neoantigen expression, which could limit the effectiveness of even personalized VV vaccines. To address this, VV platforms incorporating multiple antigens, flexible expression systems, or combination approaches with immune checkpoint inhibitors and TME modulators may be required to sustain effective, patient-specific antitumor immunity.

Together, these strategies position VV as a flexible, programmable vehicle for personalized neoantigen delivery, enabling precise stimulation of tumor-specific immunity and directly addressing the challenge of antigenic diversity in NSCLC and other thoracic malignancies.

#### 7.1.4 Barriers to systemic delivery of VV in lung cancer

Systemic administration of vaccinia virus (VV) is conceptually attractive for advanced lung cancer, where disseminated or inaccessible lesions are common. However, clinical and preclinical evidence indicates that intravenous (IV) delivery is constrained by pre-existing immunity, rapid reticuloendothelial clearance, and limited feasibility of repeat dosing (12).

A principal biological barrier is the rapid induction of neutralizing antibodies following systemic exposure. IV administration of VV consistently elicits brisk humoral responses. In a phase I study of IV vvDD, post-treatment neutralizing titers increased more than 100-fold (101, 135). Universal neutralizing responses were similarly observed after IV Pexa-Vec or intra-arterial TG6002 (26, 117, 136, 137). (236–240).

These findings indicate that systemic VV exposure reliably triggers strong antibody-mediated immunity, which may curtail circulating virus upon subsequent administration (136–140). In addition to treatment-induced responses, pre-existing anti-vaccinia immunity remains clinically relevant (90, 141).

Although routine smallpox vaccination was discontinued in 1980, individuals born before the mid-1970s may retain residual immunity. Serological surveys demonstrate that a substantial proportion of older adults maintain detectable neutralizing antibodies decades after vaccination (142, 143).

Given that lung cancer predominantly affects older patients, baseline vector immunity may meaningfully influence viral pharmacokinetics in this population. From a pharmacokinetic perspective, pre-existing humoral immunity can shorten viral circulation time following intravenous administration (69). Notably, complete prevention of tumor infection has not been consistently observed; rather, accelerated systemic clearance has been reported in immune hosts, suggesting that vaccination history functions as a quantitative modifier of viral bioavailability rather than an absolute barrier to efficacy. The impact on clinical outcomes remains uncertain, as available data do not consistently demonstrate a direct association between baseline titers and tumor response (69). Nevertheless, pre-existing immunity may constrain repeated intravenous dosing, particularly when therapeutic activity relies on sustained viremia. Prospective assessment of vaccination history and baseline neutralizing titers may therefore facilitate patient stratification and pharmacokinetic interpretation in future trials.

Beyond adaptive immunity, rapid blood clearance and reticuloendothelial sequestration further restrict systemic VV delivery. Viral genomes are detectable shortly after infusion but largely disappear within hours (101). Preclinical biodistribution studies demonstrate early localization to the liver and spleen following IV injection (144, 145), consistent with uptake by the reticuloendothelial system. Although tumor-selective replication has been documented in permissive models (146) early hepatic sequestration likely limits the fraction of virus reaching distant pulmonary or metastatic lesions. While much of this evidence derives from non–lung tumor models, these pharmacokinetic constraints are unlikely to be tumor-type specific and are therefore highly relevant to systemic strategies in lung cancer.

Collectively, these immunologic and pharmacokinetic factors restrict the feasibility of repeat intravenous administration. In murine models, a second IV dose resulted in markedly reduced or undetectable viral replication (146). Clinically, most IV VV studies have employed single-dose designs (101, 147), and robust neutralizing responses were consistently observed (141). Comprehensive human data supporting sustained systemic re-administration therefore remain limited.

Several experimental strategies are under active investigation to overcome these barriers, including regional delivery approaches, heterologous prime–boost regimens, and engineering of tumor-selective or less immunogenic viral backbones. However, definitive clinical evidence demonstrating reliable circumvention of humoral neutralization or reticuloendothelial clearance has yet to emerge.

#### 7.1.5 Rational combination approaches to enhance VV efficacy

Vaccinia virus (VV) often induces upregulation of immune checkpoints such as PD-L1, providing a rationale for combining VV with immune checkpoint inhibitors (ICIs). However, clinical results in lung cancer have been variable (40, 94, 113, 114). Reliable synergy requires more than simultaneous administration; it demands a carefully orchestrated, mechanism-driven approach.

Key strategies include mapping the spatiotemporal dynamics of the immune response after VV administration. Understanding the timing of T cell priming, checkpoint induction, and exhaustion onset allows precise scheduling and dosing of ICIs (71, 148). Combination approaches can extend beyond PD-1/PD-L1 blockade, incorporating STING or TLR agonists to boost innate sensing, metabolic modulators to counteract TME constraints, or immunogenic chemoradiotherapy tailored to lung cancer subtypes (99).

Predictive biomarkers are essential for guiding these strategies. Integrated measures capturing baseline TME composition, immune cell dynamics during treatment, soluble factor profiles, and viral activity support patient stratification and therapy monitoring (149, 150). Safety considerations, particularly the risk of immune-related pneumonitis, must inform trial design (151).

Adaptive and basket trial designs offer flexible platforms to test VV as a programmable immunological backbone rather than a simple adjunct. These designs allow multi-modal combinations to be evaluated across biologically defined lung cancer subsets, helping identify the most effective integrated regimens.

**Figure 5:**
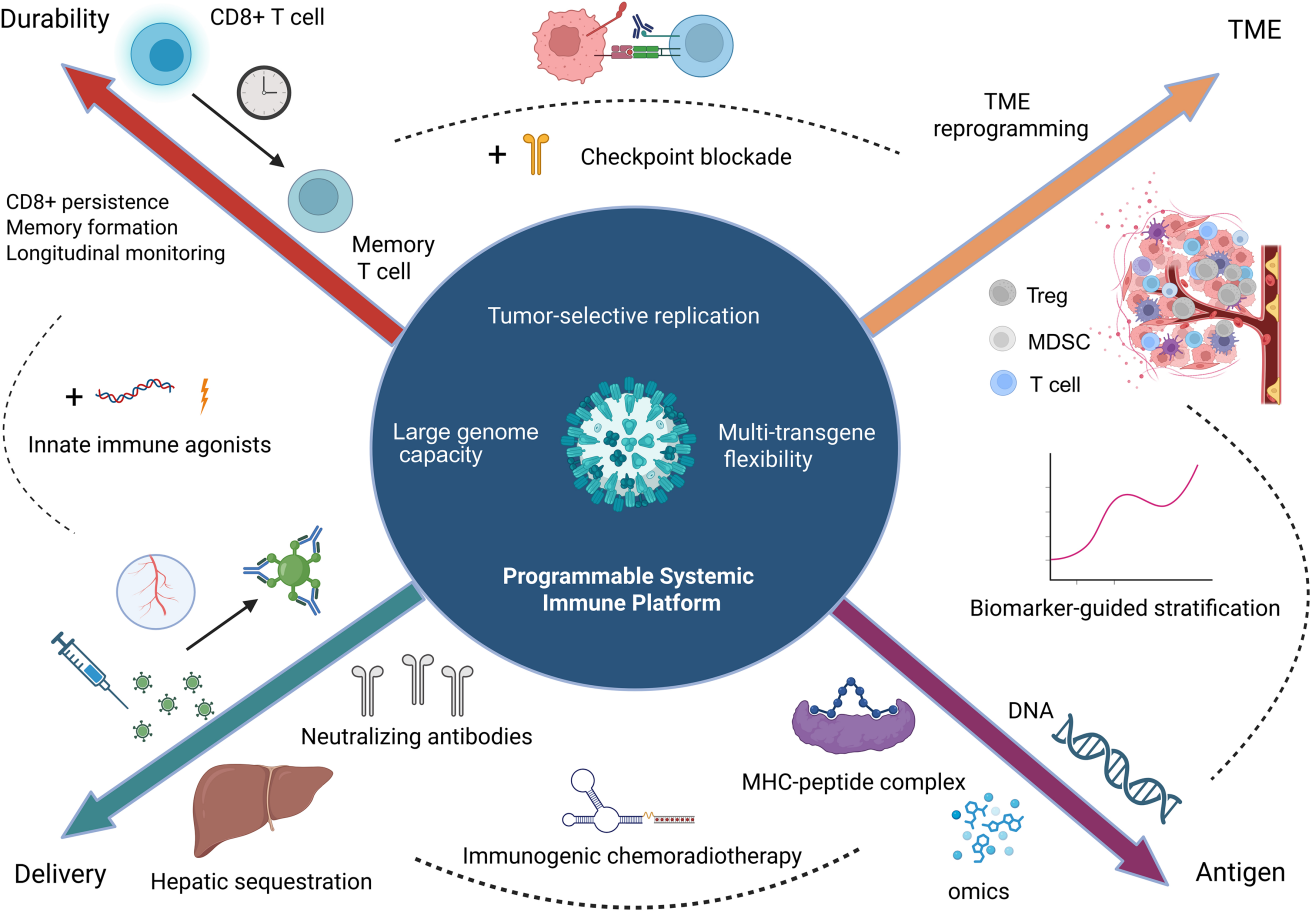
Multiaxial translational framework for programmable VV, vaccinia virus immunotherapy in NSCLC. Vaccinia virus is depicted at the center as a programmable systemic immunotherapy platform. Four axes represent key translational challenges and mechanistic considerations: Durability (CD8^+^ T cell persistence and memory formation), Tumor Microenvironment (TAM polarization, MDSC suppression, inhibitory pathway modulation), Antigen (personalized neoantigen discovery and multi-antigen encoding), and Delivery (neutralizing antibodies, hepatic sequestration, limited repeat dosing). Cross-axis interactions highlight integrative strategies, including checkpoint blockade, innate immune agonists, immunogenic chemoradiotherapy, and biomarker-guided adaptive trial design. Collectively, the diagram illustrates how VV functions as a multi-dimensional platform for systemic immune reprogramming in lung cancer.

**Figure 5** summarizes these translational challenges and forward-looking strategies for VV-based immunotherapy in lung cancer.

## 8 Conclusion

This review summarizes the preclinical progress of vaccinia virus (VV) in the context of lung cancer therapy, including its tumor-selective oncolytic activity, immunomodulatory potential, and prospects for combination with other therapeutic modalities. Existing evidence indicates that VV demonstrates robust antitumor efficacy in both *in vitro* and animal models and can modulate the tumor microenvironment through multiple mechanisms, highlighting its potential as an immunotherapeutic platform.

However, significant barriers remain for the clinical translation of VV, including limitations in systemic delivery, the impact of pre-existing host immunity, the generation of neutralizing antibodies, optimization of dosing and safety, as well as tumor heterogeneity and the complexity of the immune microenvironment. These factors may affect VV’s efficacy and reproducibility across different patients and tumor types.

The central task moving forward is to translate concept into practice. This requires developing VV-based strategies that produce sustained systemic control, generate long-term immunologic memory, and adapt to the biological diversity of lung cancer. Achieving these goals would expand the treatment landscape and elevate VV from a niche virotherapy to a core component of multimodal immunotherapy frameworks, capable of being tailored to the needs of each patient.

Accordingly, VV should currently be regarded as an experimental immunotherapeutic strategy with developmental potential rather than a near-term clinically ready therapy. Future studies should focus on optimizing viral vector design, improving delivery strategies, integrating personalized treatment approaches, and systematically evaluating safety and immune responses to facilitate its clinical translation.
