# Supplementary material for: Vaccinia virus for lung cancer therapy: preclinical progress and prospects as a systemic immunotherapy platform
Source: Front Immunol. 2026 May 7;17:1797922. doi: 10.3389/fimmu.2026.1797922 (PMC13189763; doi:10.3389/fimmu.2026.1797922)
Supplement: Supplementary file 1 [file Table1.docx]

Supplemental Figures


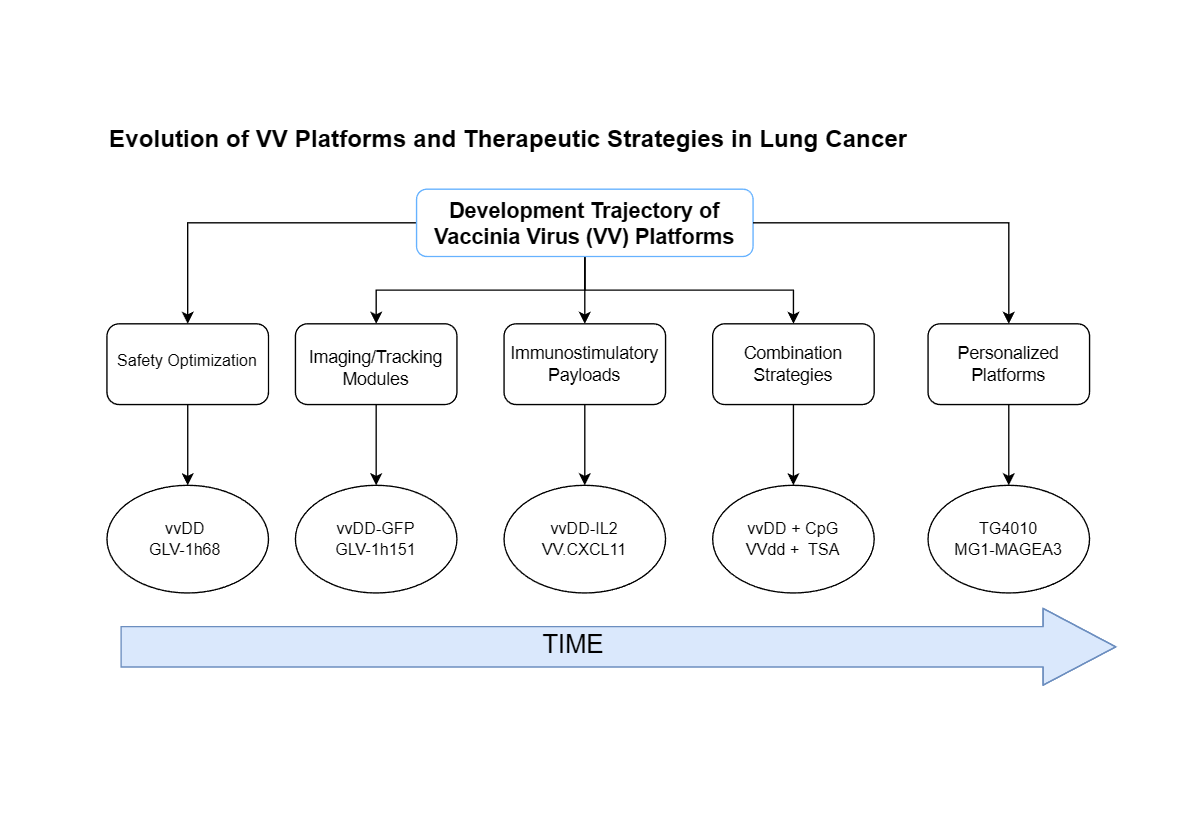


**Figure S1**. Evolution of engineered vaccinia virus platforms and therapeutic strategies in lung cancer.

A schematic timeline illustrates the progressive development of vaccinia virus (VV)-based oncolytic platforms, highlighting key milestones in their engineering and application. Initial efforts focused on safety optimization through targeted gene deletions (e.g., vvDD, GLV-1h68), enabling tumor-selective replication. Subsequent generations incorporated imaging and tracking modules (e.g., vvDD-GFP, GLV-1h151) to monitor viral biodistribution and replication in real time. Further advances included the integration of immunostimulatory payloads (e.g., IL-2, CXCL11), combination strategies with immunomodulators (e.g., CpG, TSA), and the emergence of personalized platforms (e.g., TG4010, MG1-MAGEA3). This evolution reflects a shift from foundational safety engineering toward multifunctional, tailored therapeutic systems designed to enhance antitumor immunity and treatment efficacy.


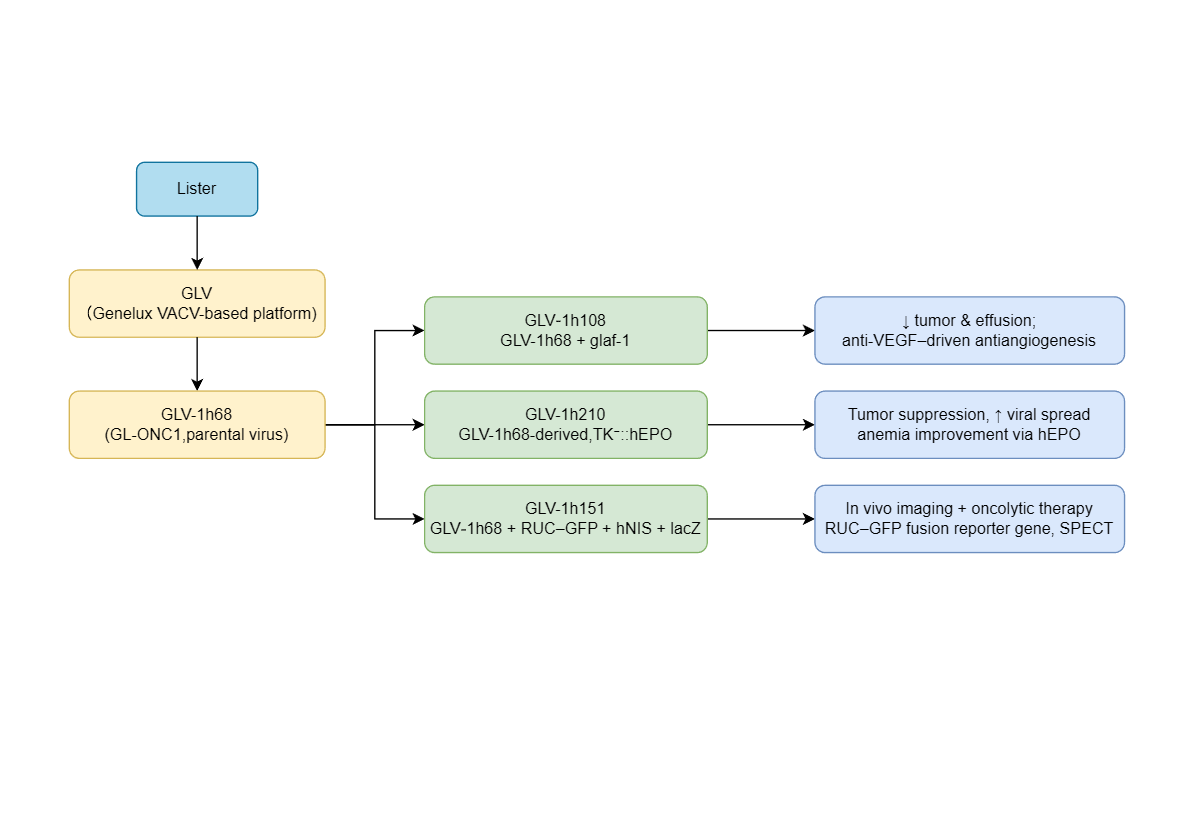


**Figure S2**. Engineering and functional enhancement of the GLV vaccinia virus platform for lung cancer therapy.

Schematic representation of the rational design and iterative optimization of GLV-based oncolytic viruses, derived from the LIVP strain. The parental virus GLV-1h68 (GL-ONC1) serves as the foundational backbone, exhibiting potent tumor suppression and inflammatory immune activation. Subsequent engineered variants include GLV-1h108, which encodes the anti-VEGF gene glaf-1 to inhibit angiogenesis and reduce malignant effusion; GLV-1h151, equipped with RUC-GFP and human sodium iodide symporter (hNIS) for real-time multimodal imaging and theranostic applications; and GLV-1h210, which expresses human erythropoietin (hEPO) to ameliorate cancer-related anemia and enhance intratumoral viral distribution. Together, these modifications illustrate a systematic progression from basic oncolytic function toward integrated diagnostic, antitumor, and supportive care capabilities.


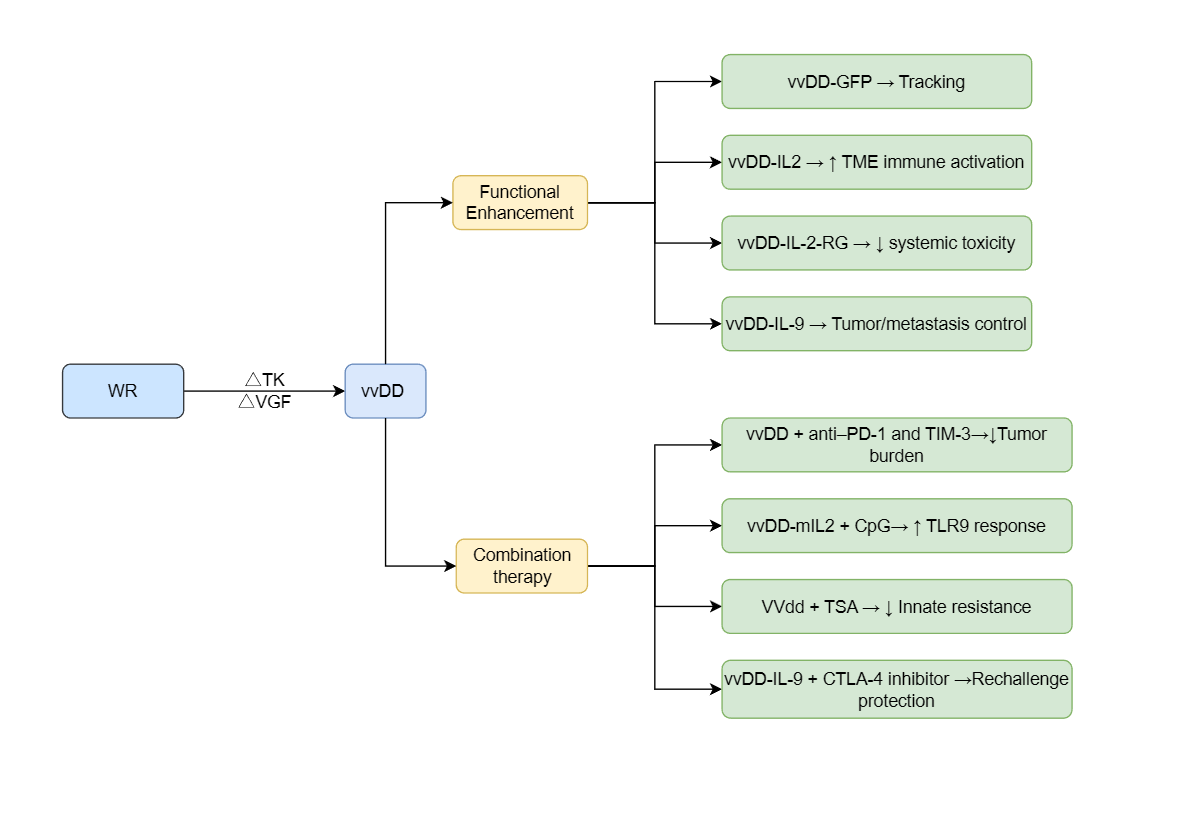


**Figure S3**. Rational engineering and combinatorial therapeutic enhancement of the vvDD vaccinia virus platform in lung cancer models.

Schematic overview of the iterative optimization of the vvDD backbone, which incorporates dual deletions of thymidine kinase (TK) and vaccinia growth factor (VGF) genes to ensure tumor-selective replication. Functional enhancements include the introduction of tracking modules (e.g., vvDD-GFP), immunostimulatory cytokines (e.g., IL-2, IL-9), and payloads designed to limit systemic toxicity (e.g., vvDD-IL-2-RG). Combination strategies with immune checkpoint inhibitors (e.g., anti-PD-1, anti–TIM-3, CTLA-4 blockade) or immunomodulators (e.g., CpG, TSA) further augment antitumor immunity, reduce tumor burden, control metastasis, and in some cases confer protection against tumor rechallenge. This systematic engineering approach underscores the transition from a replication-competent oncolytic vector toward a multimodal immunotherapeutic platform capable of remodeling the tumor microenvironment.
