# Supplementary material for: Vaccinia virus for lung cancer therapy: preclinical progress and prospects as a systemic immunotherapy platform
Source: Front Immunol. 2026 May 7;17:1797922. doi: 10.3389/fimmu.2026.1797922 (PMC13189763; doi:10.3389/fimmu.2026.1797922)
Supplement: Supplementary file 2 [file Table2.docx]

Table S1 Representative VV Strains Validated Across Multiple Research Stages

| Virus Name | Genetic Modifications | Involved Stages | Application Highlights | Key Mechanisms/Findings | PMID(s) |
| --- | --- | --- | --- | --- | --- |
| GLV-1h68^a^ | LIVP; ΔF14.5L, J2R, A56R; +RUC-GFP, LacZ, GusA | In Vitro, In Vivo, Combination | Oncolysis, immune activation, synergy with cyclophosphamide | ↑Virus titer, cytokine induction (MCP-1, TNF-α), VEGF↓, ↓VCAM-1/vWF^n^ | 32631270, 25030093, 23635329 |
| GLV-1h210^b^ | GLV-1h68-derived; TK⁻::hEPO (P7.5E promoter) | In Vitro, In Vivo | Oncolysis and anemia correction via hEPO | Time-dependent hEPO secretion, ↑tumor suppression, ↑viral replication | 23765443 |
| vvDD^c^ | WR.ΔTK, ΔVGF | In Vitro, In Vivo, Combination | Selective killing, systemic immune activation, combined immunotherapy | CD4/CD8 T cell activation, ↑apoptosis, ↓TME suppression | 32461344, 21283510, 22315352 |
| vvDD-mIL2^d^ | WR.ΔTK, ΔVGF; +membrane-bound IL-2 | In Vivo, Combination | Bilateral tumor control, immune enhancement | CpG/TLR9/anti–PD-1 co-stimulation, ↑systemic immunity | 32405533 |
| vvDD-IL-2-RG^e^ | WR.ΔTK, ΔVGF; +IL-2 (RG + GPI anchor), YFP | In Vivo, Combination | TME remodeling and checkpoint synergy | ↑CD8⁺ T cells, ↓systemic toxicity, ↑effect in high-burden tumors | 30410056 |
| vvDD-IL-9^f^ | WR.ΔTK, ΔVGF; +IL-9, YFP | In Vivo, Combination | CD8⁺ CCR6⁺ cell recruitment, checkpoint synergy | CTLA-4 blockade synergy, tumor rechallenge protection | 38473379 |
| VV.CXCL11^g^ | WR.ΔTK, ΔVGF; +murine CXCL11, luc | In Vivo, Combination | Combination with vaccine / CAR-T | ↑Ag-specific CD8⁺ T cells, TME reprogramming | 29399394 |
| VV.mIFNβ^h^ | WR.ΔTK, ΔB18R; +mIFNβ, luc | In Vivo, Combination | Oncolysis, T-cell priming, vaccine synergy | ↑CD8⁺ T cell activation, ↑IFNβ expression, enhanced regression | 22008913 |
| TG6050^i^ | Copenhagen.ΔTK/RR/M2L; +hu-IL-12 + anti–CTLA-4 | In Vivo, Combination | Immune remodeling, checkpoint combination | ↑CD8⁺ T cells, ↓Treg, ↑M1 macrophages | 11284822 |
| EphA2-TEA-VV^j^ | WR.VSC20(ΔTK, ΔVGF); +EphA2-TEA bispecific engager | In Vitro, In Vivo, Combination | Dual targeting of tumor and T cells, systemic immunity | Local engager secretion, ↑bystander killing, ↓lung metastasis | 24135899 |
| VVΔTKΔN1L^k^ | WR or Lister (context-dependent); ΔTK, ΔN1L | In Vivo, Combination | Neoadjuvant therapy; innate immunity–dependent effect | NK cell–mediated efficacy; independence from adaptive immunity | 32217766 |
| VVL-m12^l^ | ListerΔTK/ΔN1L; IL-12 knock-in | In Vivo, Combination | TME remodeling, PD-1 blockade synergy | ↑CD8⁺, ↓Treg, long-term memory, >90% CR with PD-1 blockade | 39840061 |
| vB7/β/IL-12^m^ | +B7-1, IL-12, lacZ, gpt | In Vivo, Combination | Lung metastasis suppression, IL-12 synergy | Enhanced T cell responses with exogenous IL-12 | 9862625 |

**Footnotes**
a GLV-1h68: LIVP backbone; F14.5L, J2R, A56R deletions; RUC-GFP, LacZ, GusA reporters
b GLV-1h210: GLV-1h68-derived, TK deletion, hEPO inserted under P7.5E promoter
c vvDD: WR backbone, TK and VGF deletions
d vvDD-mIL2: vvDD with membrane-bound IL-2 insertion
e vvDD-IL-2-RG: vvDD with IL-2 fused to RG domain and GPI anchor, with YFP reporter
f vvDD-IL-9: vvDD with IL-9 insertion, YFP reporter
g VV.CXCL11: vvDD with murine CXCL11 insertion, luciferase reporter
h VV.mIFNβ: WR backbone, TK and B18R deletions, murine IFNβ insertion, luciferase reporter
i TG6050: Copenhagen backbone, TK/RR/M2L deletions; hu-IL-12 + anti–CTLA-4 insertion
j EphA2-TEA-VV: WR.VSC20 backbone (TK and VGF deletions), EphA2-CD3 bispecific engager insertion
k VVΔTKΔN1L: WR or Lister backbone, TK and N1L deletions
l VVL-m12: Lister backbone, TK and N1L deletions, IL-12 knock-in
m vB7/β/IL-12: B7-1, IL-12, LacZ, gpt insertions
n ↑, increase or enhancement; ↓, decrease or reduction

**Abbreviations:**
TK, thymidine kinase; VGF, vaccinia growth factor; RR, ribonucleotide reductase; M2L, host range/immune modulation gene; N1L, NF-κB inhibitory gene; B18R, soluble IFNα/β receptor; GFP, green fluorescent protein; YFP, yellow fluorescent protein; LacZ, β-galactosidase; hNIS, human sodium iodide symporter; RUC, Renilla luciferase; luc, luciferase; GusA, β-glucuronidase; hEPO, human erythropoietin; P7.5E, P7.5 early promoter; hu-IL-12, human interleukin-12; mIFNβ, murine interferon beta; CXCL11, C-X-C motif chemokine ligand 11; CpG, cytosine-phosphate-guanine oligodeoxynucleotide; TLR9, Toll-like receptor 9; TEA, T-cell engaging antibody; CR, complete response; TME, tumor microenvironment; PD-1, programmed cell death-1; CTLA-4, cytotoxic T-lymphocyte–associated protein 4; NK, natural killer cell; MCP-1, monocyte chemoattractant protein-1; TNF-α, tumor necrosis factor alpha; VEGF, vascular endothelial growth factor; VCAM-1, vascular cell adhesion molecule 1; vWF, von Willebrand factor; CD4⁺/CD8⁺ T cells, CD4/CD8-positive T lymphocytes; Treg, regulatory T cell; M1 macrophages, classically activated macrophages; Ag, antigen; GPI, glycosylphosphatidylinositol anchor; gpt, guanine phosphoribosyltransferase.
